# Photocatalytic Semi‐Hydrogenation of Acetylene to Polymer‐Grade Ethylene with Molecular and Metal–Organic Framework Cobaloximes

**DOI:** 10.1002/adma.202408658

**Published:** 2024-10-22

**Authors:** Aaron E.B.S. Stone, Anna Fortunato, Xijun Wang, Edoardo Saggioro, Randall Q. Snurr, Joseph T. Hupp, Francesca Arcudi, Luka Ðorđević

**Affiliations:** ^1^ Department of Chemistry Northwestern University 2145 Sheridan Rd. Evanston IL 60208–3113 USA; ^2^ Department of Chemical Sciences University of Padova Via F. Marzolo 1 Padova 35131 Italy; ^3^ Department of Chemical and Biological Engineering Northwestern University 2145 Sheridan Rd. Evanston IL 60208–3120 USA

**Keywords:** acetylene reduction, hydrogenation, metal–organic frameworks, molecular catalysts, photocatalysis

## Abstract

The semi‐hydrogenation of acetylene in ethylene‐rich gas streams is a high‐priority industrial chemical reaction for producing polymer‐grade ethylene. Traditional thermocatalytic routes for acetylene reduction to ethylene, despite progress, still require high temperatures and high H_2_ consumption, possess relatively low selectivity, and use a noble metal catalyst. Light‐powered strategies are starting to emerge, given that they have the potential to use directly the abundant and sustainable solar irradiation, but are ineffective. Here an efficient, >99.9% selective, visible‐light powered, catalytic conversion of acetylene to ethylene is reported. The catalyst is a homogeneous molecular cobaloxime that operates in tandem with a photosensitizer at room temperature and bypasses the use of non‐environmentally friendly and flammable H_2_ gas feed. The reaction proceeds through a cobalt‐hydride intermediate with ≈100% conversion of acetylene under competitive (ethylene co‐feed) conditions after only 50 min, and with no evolution of H_2_ or over‐hydrogenation to ethane. The cobaloxime is further incorporated as a linker in a metal–organic framework; the result is a heterogeneous catalyst for the conversion of acetylene under competitive (ethylene co‐feed) conditions that can be recycled up to six times and remains catalytically active for 48 h, before significant loss of performance is observed.

## Graphical abstract

Novel photocatalytic systems based on homogeneous and heterogeneous cobaloximes are reported. By leveraging their hydrogen atom transfer catalytic activity, exquisitely efficient and selective acetylene photocatalytic semi‐hydrogenation is reported. Integrating the cobaloximes in a metal–organic framework results in more stable and durable catalysis.

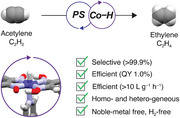

## 1 Introduction

Ethylene is one of the world's most important commodity chemicals. ≈200 million tons are produced annually, the most of any organic compound, with ethylene being an intermediate in 50–60% of all plastics.^[^ ^1^ ^]^ The vast majority of ethylene is derived from the steam cracking of petroleum, a process that unavoidably introduces impurities into the crude ethylene stream.^[^ ^1^, ^2^ ^]^ From the standpoint of plastics production, the most pernicious of these contaminants is acetylene, which poisons the Ziegler‐Natta catalysts that polymerize ethylene into plastics.^[^ ^1^, ^2^, ^3^ ^]^ Typically, crude ethylene streams contain 0.5‐2% acetylene by volume; prior to plastics production, removal of the acetylene impurity is imperative.^[^ ^1^, ^3^ ^]^

The current industrial standard for thermocatalytic acetylene semi‐hydrogenation has weaknesses in that it uses high temperatures and pressures, H_2_ co‐feed, and precious metal Pd catalysts.^[^ ^4^ ^]^ This reaction is additionally prone to over‐hydrogenation to ethane, a less valuable product that also inhibits polymerization of ethylene, and therefore must be removed using cryogenic sublimation prior to plastics production.^[^ ^5^ ^]^ The use of Pd and excess H_2_ as co‐feed limits the ethylene selectivity of the thermocatalytic process to 85% with >90% conversion at 200 °C.^[^ ^2^, ^3^ ^]^ Recently, physisorption‐based acetylene purification^[^ ^6^ ^]^ or electro‐^[^ ^7^ ^]^ and photo‐catalytic routes have been pursued, by our research groups and others, to avoid the drawbacks of the thermochemical routes.^[^ ^3^ ^]^ We sought to improve upon selectivity and conversion by designing photocatalytic systems based on Co‐porphyrin catalysts, and succeeded in creating both a homogenous system based on [*meso*‐tetra(4‐sulfonatophenyl)porphyrinato]‐cobalt(III)^[^ ^8^ ^]^ and a heterogeneous system based on the metal–organic framework (MOF) Co‐PCN‐222 (with [*meso*‐tetra(4‐carboxyphenyl)porphyrinato]‐cobalt(III) linkers).^[^ ^9^ ^]^ Notably, both achieved ≈100% conversion with >99% selectivity for ethylene over ethane under industrially relevant conditions (dilute acetylene in a predominantly ethylene stream). However, long irradiation times (>28 h) with both homogenous and heterogenous Co‐porphyrins systems are necessary to achieve photocatalytic conversion of acetylene under a mixed acetylene/ethylene atmosphere (the industrially relevant conditions), which makes it unlikely for the photocatalytic process to be implemented industrially. Therefore, the discovery of novel, more efficient catalysts, and their integration in photocatalytic systems for this industrially relevant transformation remain essential. Combining the high activity, selectivity, and tunability of homogeneous catalysts with the durability and recyclability of heterogeneous catalysts represents a grand achievement in catalysis. Metal–organic frameworks (MOFs) are excellent candidates for achieving this goal through a molecular approach to heterogeneous catalysis. MOFs are a class of porous, crystalline, multi‐dimensional nanomaterials composed of inorganic metal ions or clusters (nodes) connected by polytopic organic molecules (linkers).^[^ ^10^ ^]^ Pursuing the use of MOFs in catalysis can offer some additional advantages, such as: i) their chemical stability and high surface areas make them great supports for highly dispersed and well‐isolated catalysts;^[^ ^11^ ^]^ ii) the plethora of choices for both the inorganic and organic components of MOFs, allowing for the incorporation of molecular catalysts into the framework as either the node^[^ ^12^ ^]^ or the linker;^[^ ^13^ ^]^ iii) MOFs are also prized for the ease with which they can be post‐synthetically modified, which allows catalysts to be installed and distributed throughout the framework after synthesis;^[^ ^14^ ^]^ iv) the crystallinity and long‐range order of MOFs make it possible, in principle, to engineer the same microenvironment for all catalyst species to achieve true single‐site catalysis.^[^ ^15^ ^]^

**Figure 1:**
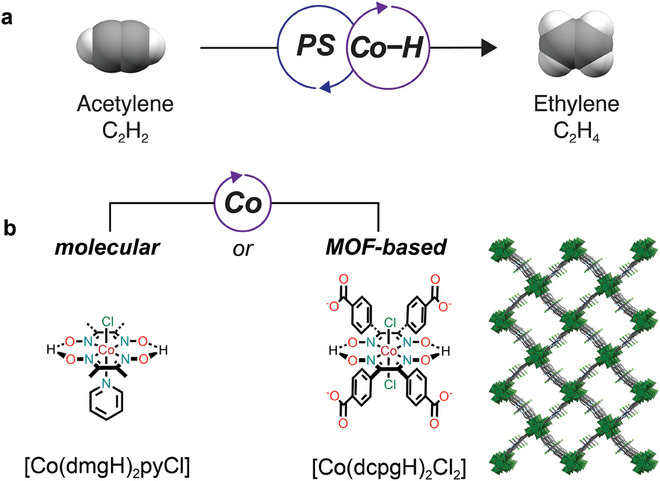
Photocatalytic semi‐hydrogenation of acetylene by cobaloxime‐based catalysts through cobalt‐catalyzed hydrogen atom transfer reaction. a) The photocatalytic semi‐hydrogenation of C_2_H_2_ using cobaloxime‐based photocatalytic systems can be achieved using b) either a homogeneous molecular approach or a heterogeneous MOF‐based approach (structural model as reported^[^ ^20^ ^]^).

In the previously reported photocatalytic reaction conditions for acetylene semi‐hydrogenation with cobalt porphyrins, the hydrogenation was proposed to proceed through the acetylene π‐complex pathway,^[^ ^8^ ^]^ which could limit the activity due to the protonation of the π‐complex being the rate determining step of the catalytic cycle.^[^ ^16^ ^]^ An alternative route to alkyne hydrogenation could be a hydrogen atom transfer (HAT) reaction, through a metal hydride intermediate.^[^ ^17^ ^]^ We therefore hypothesized that catalysts capable of performing HAT reactions via a Co^III^–H intermediate would be good candidates for photocatalytic acetylene hydrogenation (**Figure** **1**a). Therefore, to improve upon Co‐porphyrin‐based systems, and to emphasize the ability of MOFs to stabilize delicate homogeneous catalysts, here we pursue cobaloxime‐based systems for the photocatalytic semi‐hydrogenation of acetylene (Figure 1b). Cobaloximes are octahedral Co complexes composed of two equatorial dioxime ligands, commonly connected by hydrogen bonding (Figure 1b). The upward axial ligand is typically a halide, while the downward axial ligand can be either another halide, or a nitrogen base such as pyridine.^[^ ^18^ ^]^ The relative ease of preparation, together with synthetic flexibility and tunability of the ligand structure, have made cobaloximes a popular choice for development of photocatalytic systems.^[^ ^19^ ^]^ Cobaloximes are among the most widely studied molecular catalysts for hydrogen evolution, exhibit excellent activity, and can be paired with numerous organic and inorganic photosensitizers.^[^ ^18^, ^20^ ^]^ Furthermore, it is well‐established that cobaloxime‐mediated H_2_ evolution proceeds through a Co^III^–H intermediate.^[^ ^18^, ^20^ ^]^ Cobalt hydride intermediates have been also reported for HAT reaction to organic substrates,^[^ ^21^ ^]^ which suggests that this method could be extended to acetylene semi‐hydrogenation. As they exist in the same phase as the reaction mixture, separation and reuse of molecular cobaloximes from the reaction mixture can be, however, extremely difficult or impossible. Furthermore, molecular cobaloximes are known for their long‐term instability due to only hydrogen bonds holding together the equatorial ligand framework.^[^ ^18^, ^20^ ^]^ To stabilize the cobaloxime motifs, the following strategies can be pursued: i) excess ligand is often added to stabilize the cobaloximes in catalytic studies;^[^ ^22^ ^]^ ii) the internal hydrogen bonding of the oximes can be replaced by difluoroborylate (BF_2_) groups;^[^ ^19^, ^20^ ^]^ and iii) cobaloximes can be stabilized by incorporation into a MOF.^[^ ^14^, ^20^ ^]^

Here, we report two novel photocatalytic systems for the semi‐hydrogenation of acetylene to ethylene: one homogeneous system using a molecular cobaloxime and one heterogeneous system based on a metal–organic framework with a cobaloxime linker (Figure 1b). The catalysts are photosensitized by tris(2,2′‐bipyridyl)ruthenium(II) chloride ([Ru(bpy)_3_]^2+^). We first developed a catalytic system based on the molecular catalyst chloro(pyridine)bis(dimethylglyoximato)cobalt(III) ([Co(dmgH)_2_pyCl], dmgH = dimethylglyoxime, py = pyridine), which exhibits excellent catalytic activity for the conversion of acetylene to ethylene with turnover number (TON) = 1148 and >99.9% selectivity for ethylene versus ethane after 20 h of visible‐light irradiation (450 nm). The homogeneous system is also highly competent for ethylene purification (industrially relevant conditions), achieving ≈100% visible‐light powered conversion of acetylene to ethylene in just 50 min, with no evidence of over‐hydrogenation to ethane, such that the selectivity for ethylene versus ethane is >99.9%. Detailed mechanistic studies reveal that acetylene semi‐hydrogenation relies on production of a Co^I^ active species that is protonated to form a Co^III^–H intermediate, as anticipated. The homogeneous system demonstrates limited reusability under the industrial mixture conditions, and the catalytic solution stops working after three re‐uses. To increase recyclability and to improve the durability of our system, we translated our homogeneous system into a heterogeneous one by incorporating molecular cobaloxime as linkers in a MOF. The heterogeneous system displays similarly fast reduction kinetics and same excellent selectivity for acetylene semi‐hydrogenation as the homogeneous system. Critically, the MOF‐based system retains catalytic activity for more than twice as long as the homogeneous system (48 h of irradiation) and can be recycled twice as many times before performance deteriorates.

## 2 Results and Discussion

### 2.1 Chloro(Pyridine)Cobaloxime(III) Exhibits Excellent Activity and Selectivity for the Photocatalytic Semi‐hydrogenation of Acetylene to Ethylene

#### 2.1.1 Non‐Competitive Conditions (Pure Acetylene, no Ethylene Co‐Feed)

**Figure 2:**
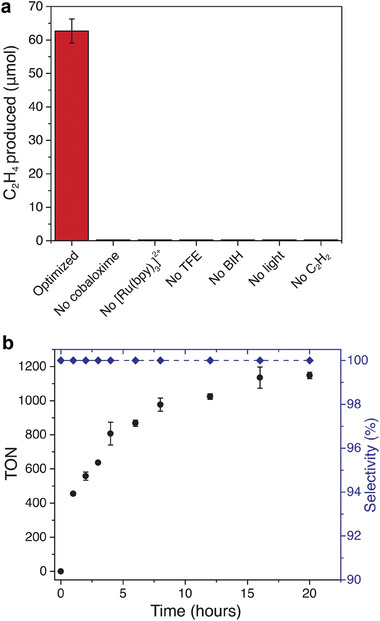
Performance of the homogeneous cobaloxime‐based photocatalytic system for the semi‐hydrogenation of acetylene to ethylene under noncompetitive conditions (pure acetylene, no ethylene co‐feed). a) Total C_2_H_4_ production for the optimized reaction mixture containing 2.5 mm [Ru(bpy)_3_]^2+^, 1.0 mm [Co(dmgH)_2_pyCl], 1.0 m TFE and 0.1 m BIH in acetonitrile irradiated (450 nm, 140 mW∙cm^−2^) for 4 h under C_2_H_2_ (≥99.5 vol.%), and for reaction mixtures that differ from the optimized conditions as indicated by the axis labels. b) TON (C_2_H_4_) and C_2_H_4_ selectivity over C_2_H_6_ as a function of irradiation time (450 nm) for the [Ru(bpy)_3_]^2+^/[Co(dmgH)_2_pyCl] system containing 2.5 mm [Ru(bpy)_3_]^2+^, 1.0 µm [Co(dmgH)_2_pyCl], 1.0 m TFE and 0.1 m BIH in acetonitrile under C_2_H_2_ (≥99.5 vol.%). Error bars represent the standard deviations for three separate experiments.

Our photocatalytic system consists of four components: [Co(dmgH)_2_pyCl] as catalyst, [Ru(bpy)_3_]^2+^ as photosensitizer (PS), 2,2,2‐trifluoroethanol (TFE) as proton source, and 1,3‐dimethyl‐2‐phenyl‐2,3‐dihydro‐1*H*‐benzo[*d*]imidazole (BIH) as sacrificial donor (SD). In a typical experiment, we illuminated the catalytic mixture in acetonitrile under 1 atm C_2_H_2_ (≥99.5 vol.%) using a 450 nm light‐emitting diode (LED, 140 mW∙cm^−2^); the details of the purging and photocatalytic setups are published elsewhere.^[^ ^8^ ^]^ Crucially, a typical gas chromatogram and mass spectrum (Figure S1, Supporting Information) of the reaction mixture, after 4 h of irradiation, shows conversion of C_2_H_2_ to C_2_H_4_ without any appreciable over‐hydrogenation to C_2_H_6_ based on the limits of detection of our instruments (calibration curves are shown in Figures S2 and S3, Supporting Information). We chose BIH and TFE in an attempt to create mild photocatalytic conditions for our cobaloxime catalyst and due to previous reports touting their excellence as SD^[^ ^14^, ^22^, ^23^ ^]^ and proton source,^[^ ^24^ ^]^ respectively. A combination of BIH and TFE showed the highest catalytic activity in a screening of various combinations (Figure S4, Supporting Information). Based on optimization of the system (Figures S5 and S6, Supporting Information), we chose concentrations for each of our reaction components: 2.5 mm [Ru(bpy)_3_]^2+^, 1.0 m TFE, 0.1 m BIH, and either 1.0 µm [Co(dmgH)_2_pyCl] to maximize turnover number (TON) or 1.0 mm [Co(dmgH)_2_pyCl] to maximize conversion of C_2_H_2_. Illumination for 4 h of the photocatalytic mixture containing 1.0 mm [Co(dmgH)_2_pyCl] produced 63 µmol C_2_H_4_ (**Figure** **2**a) with a quantum yield (QY) of 1.0%, a 10‐ and 50‐fold improvement over our recently investigated Co‐porphyrin (QY = 0.11%)^[^ ^8^ ^]^ or Co‐PCN‐222 system (QY = 0.02%),^[^ ^9^ ^]^ respectively. In the absence of [Co(dmgH)_2_pyCl], [Ru(bpy)_3_]^2+^, TFE, BIH, light, or C_2_H_2_, the C_2_H_2_ reduction does not occur, which confirms the photocatalytic nature of the reaction (Figure 2a). Using 1.0 µm [Co(dmgH)_2_pyCl], we obtain TON (C_2_H_4_) >1000 (TON = 1148) with selectivity for ethylene over ethane >99.9% after 20 h of illumination (Figure 2b). We detected no other gaseous products, except for an extremely small amount of H_2_ (2 nmol of H_2_ were generated after 4 h). As a result, the overall selectivity for C_2_H_4_ versus C_2_H_6_ and H_2_ combined is >99.9%.

Depending on the initial concentration of cobaloxime, ethylene production saturates for different reasons. At high cobaloxime concentrations (1.0 mm), catalysis stops due to depletion of BIH, as evidenced by re‐addition experiments, in which we first irradiated the system for 20 h, then selectively re‐added various components of the reaction mixture, and irradiated for an additional 4 h. These experiments show that it is possible to restart catalysis only upon addition of BIH and no other component of the reaction mixture (Figure S7, Supporting Information). At low cobaloxime concentrations (1.0 µm), however, where the available supply of BIH is not exhausted over the course of the reaction, catalysis most likely ceases due to degradation of the catalyst. Re‐addition of [Co(dmgH)_2_pyCl] restarts catalysis most effectively compared to re‐addition of other reaction components (Figure S8, Supporting Information). The delicate nature of cobaloxime catalysts has been reported previously,^[^ ^20^, ^22^ ^]^ and our own experiments showed enhanced C_2_H_4_ production in the presence of excess dimethylglyoxime ligand (Figure S9, Supporting Information).

The photocatalytic activity of our [Co(dmgH)_2_pyCl]‐based system is not limited to the semi‐hydrogenation of acetylene. Illumination for 24 h of our [Ru(bpy)_3_]^2+^/[Co(dmgH)_2_pyCl] system and phenylacetylene produces styrene (Figure S10, Supporting Information) thus showing that our system is competent to expand the scope of alkyne semi‐hydrogenation reactions.

#### 2.1.2 Competitive Conditions (Ethylene Co‐Feed)

**Figure 3:**
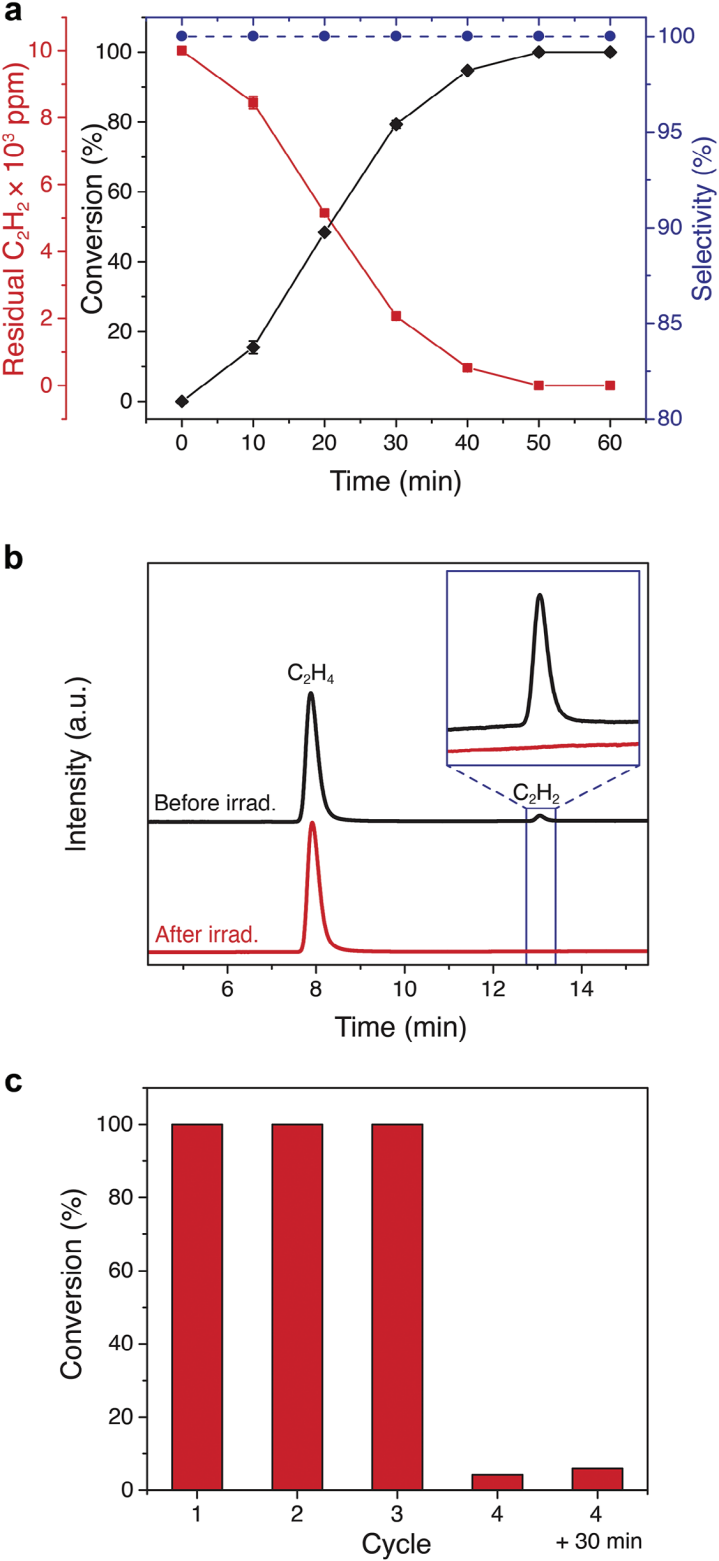
Performance of the homogeneous system under industrially relevant conditions (1 vol.% C_2_H_2_, 30 vol.% C_2_H_4_, He balance). a) Plot of C_2_H_2_ conversion (%), residual C_2_H_2_ (ppm), and C_2_H_4_ selectivity versus C_2_H_6_ (%) as a function of irradiation time. b) Gas chromatograms (the elution order is C_2_H_4_, C_2_H_6_, C_2_H_2_) detected with flame ion detection before and after irradiation (450 nm) for 50 min. The inset is a magnified view of the C_2_H_2_ peak in the chromatogram before and after illumination. c) Reuse of the photocatalytic system under industrial mixture conditions.

Critically, our [Co(dmgH)_2_pyCl]‐based system selectively reduces C_2_H_2_ to C_2_H_4_ even in the presence of excess C_2_H_4_ (1 vol.% C_2_H_2_, 30 vol.% C_2_H_4_, He balance). This C_2_H_4_/C_2_H_2_ mixture represents a typical industrially relevant ethylene feed, and a highly selective catalyst is required to avoid over‐hydrogenation to C_2_H_6_. The performance of our system under industrially relevant mixture conditions is shown in **Figure** **3**. Not only does our system reduce the C_2_H_2_ concentration from 10 000 ppm to undetectable levels without any observed over‐hydrogenation to C_2_H_6_ (Figure 3a,b; Figure S11, Supporting Information), it does so in less than 1 h (50 min), which is a 30‐ and 100‐fold improvement over our recently investigated Co‐porphyrin (28 h)^[^ ^8^ ^]^ or Co‐PCN‐222 system (87 h),^[^ ^9^ ^]^ respectively. Interestingly, the [Co(dmgH)_2_pyCl]‐based system efficiently and selectively reduces the C_2_H_2_ concentration from 10000 ppm to undetectable levels with 99.8% ethylene selectivity over ethane even in the presence of 99% C_2_H_4_ (1 vol.% C_2_H_2_, 99 vol.% C_2_H_4_), and it does so in half an hour (34 min) (Figure S12, Supporting Information). Importantly, the calculated production efficiency of our system is ≈13 L g^−1^ h^−1^ (liters of ethylene produced over gram of catalyst per hour), which is a factor of ≈7 higher than the previously investigated homogeneous batch Co‐porphyrin system.^[^ ^8^ ^]^ Our batch‐based production efficiency exceeds the industrial demand of >10 L g^−1^ h^−1^,^[^ ^7^ ^]^ making its industrial implementation promising and could be even further improved in a flow photochemical set‐up.^[^ ^25^ ^]^

The excellent performance of our system under the industrially relevant conditions allows for its reuse. The same catalyst solution can be purged with fresh C_2_H_4_/C_2_H_2_ mixture up to three times before losing its ability to reduce all C_2_H_2_ in the headspace to C_2_H_4_ (Figure 3c), most likely due to its low structural stability that is caused by the fragile ligands around the Co center where the individual oximes are only held together by weak hydrogen bonding (Figures S8 and S9, Supporting Information). Our transfer hydrogenation reaction of acetylene by taking TFE as hydrogen source is a promising alternative over conventional hydrogenation technology with a flammable H_2_ atmosphere. When the photoreduction was instead performed using deuterated TFE as deuterium source, we observed the formation of C_2_D_4_ (*m*/*z*  =  32) from C_2_D_2_ (*m*/*z*  =  28) (Figure S13, Supporting Information). C_2_D_2_ is produced by exchange between the feedstock C_2_H_2_ and deuterated TFE, which we pre‐equilibrated before illumination. Then, the addition of two deuterium to C_2_D_2_ yields C_2_D_4_. These two experiments prove that acetylene is the precursor for the observed C_2_H_4_ and that the protons added to make the C_2_H_4_ reduction product originate from TFE. Additionally, we show that water can be the source of protons for the reaction (Figure S4, Supporting Information), which is advantageous from a sustainability standpoint as it avoids the use of an organic, fossil‐fuel derived proton source.^[^ ^26^ ^]^

### 2.2 Proposed Mechanism

**Figure 4:**
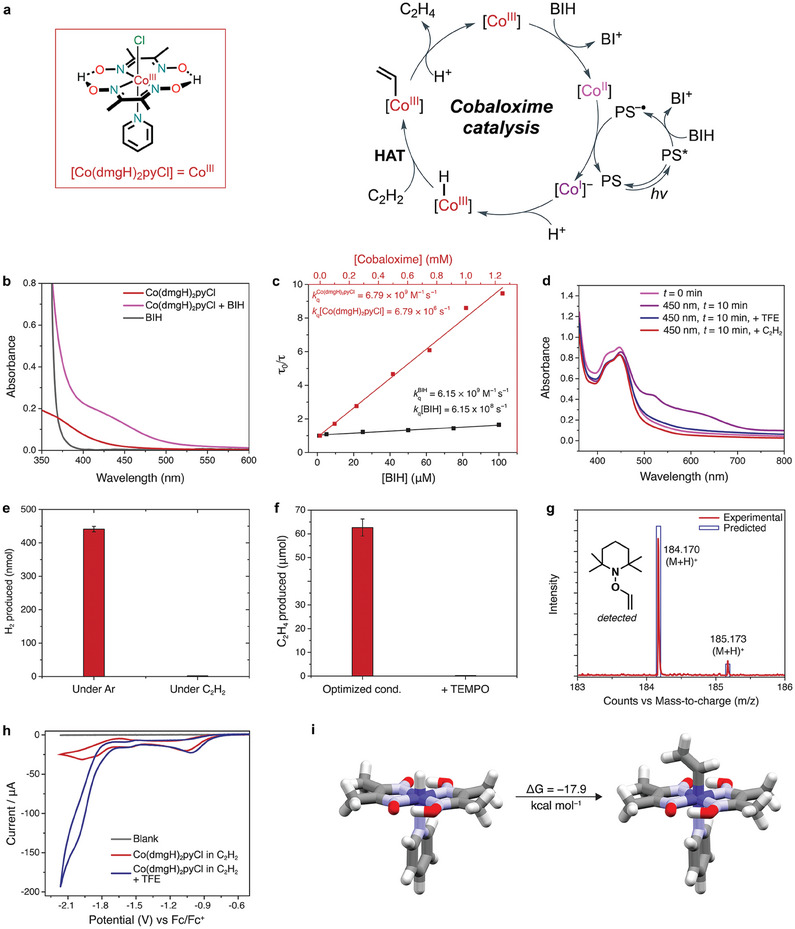
Proposed mechanism of C_2_H_2_ semi‐hydrogenation. a) Proposed catalytic mechanism for the photocatalytic reduction of C_2_H_2_ to C_2_H_4_ using [Co(dmgH)_2_pyCl]. b) UV–vis spectra of 90 mm BIH (black) and 100 µm [Co(dmgH)_2_pyCl] without (red) and with (magenta) 90 mm BIH. c) Stern‐Volmer plots for the quenching of [Ru(bpy)_3_]^2+^ emission lifetime by [Co(dmgH)_2_pyCl] (red) or BIH (black). d) UV–vis spectra of the system containing 100 µm [Co(dmgH)_2_pyCl], 50 µm [Ru(bpy)_3_]^2+^ and 85 mm BIH under various conditions (1 M TFE or ≥99.5 vol.% C_2_H_2_). e) H_2_ evolution by the [Co(dmgH)_2_pyCl]‐based system under Ar versus C_2_H_2_. f) Comparison of C_2_H_4_ production by the [Co(dmgH)_2_pyCl]‐based system under optimized conditions and in the presence of 200 molar equiv. TEMPO with respect to [Co(dmgH)_2_pyCl]. Error bars represent the standard deviations for three separate experiments. g) Detection of the TEMPO‐acetylene adduct using LCMS. h) Cyclic voltammograms of [Co(dmgH)_2_pyCl] without (red) and with (blue) TFE in acetonitrile (TBA•PF_6_ as supporting electrolyte) and saturated with C_2_H_2_. i) Density functional theory optimized structures for cobaloxime‐hydride and cobaloxime‐vinyl complexes involved in the HAT reaction.

Based on our experimental results and the literature, we propose a mechanism for the photocatalytic semi‐hydrogenation of C_2_H_2_ to C_2_H_4_ using our system in **Figure** **4**a. The addition of BIH (90 mm) to a solution of [Co(dmgH)_2_pyCl] (100 µm) in acetonitrile results in the appearance of a new absorption peak at 423 nm in the UV–vis spectrum, indicative of a Co^II^ species (Figure 4b).^[^ ^22^, ^27^ ^]^ In order to detect the formation of paramagnetic Co(II) we used electron paramagnetic resonance (EPR) spectroscopy, which showed the EPR signal^[^ ^28^ ^]^ from [Co^II^(dmgH)_2_pyCl]^−^ as a result of the reduction of [Co^III^(dmgH)_2_pyCl] in presence of BIH (Figure S14, Supporting Information). BIH can therefore chemically reduce [Co(dmgH)_2_pyCl] from Co^III^ to Co^II^, and we speculate that this is another reason why re‐addition of BIH results in the recovery of catalytic activity at high cobaloxime concentrations. Following light absorption by [Ru(bpy)_3_]^2+^ (PS), the emission of the excited state [Ru(bpy)_3_]^2+*^ photosensitizer (PS*) is quenched by hole transfer to BIH to form [Ru(bpy)_2_(bpy^•−^)]^+^ (PS^•−^), followed by electron transfer from PS^•−^ (reduction potential of −1.33 V vs SCE in acetonitrile)^[^ ^29^ ^]^ to the Co^II^ (Co^II^/Co^I^ reduction potential of −1.13 V vs SCE in acetonitrile)^[^ ^20^, ^27^ ^]^ to form the low valent Co^I^ species. We observed that the unimolecular quenching rate constant for BIH exceeds that of [Co(dmgH)_2_pyCl] by a factor of ≈ 100 according to Stern‐Volmer analysis (Figure 4c). As a result, we conclude that [Ru(bpy)_3_]^2+^ photoluminescence is reductively quenched in the presence of BIH, due to the potency of BIH as an electron donor, and thus exclude an oxidative quenching of the sensitizer by the catalyst.

We found direct spectroscopic evidence for a reactive Co^I^ intermediate in UV–vis studies of our system. Irradiation of a solution containing [Co(dmgH)_2_pyCl], [Ru(bpy)_3_]^2+^, and BIH results in the rapid formation of two new absorption peaks at 530 and 656 nm in the UV–vis spectrum (Figure 4d). These peaks have previously been assigned to the formation of a Co^I^ intermediate.^[^ ^22^, ^30^ ^]^ Upon either i) purging this solution with C_2_H_2_ or ii) adding 1.0 m TFE, the Co^I^ signature peaks disappear, and the original absorption profile of Co^III^ complex is recovered (Figure 4d). Taken together, these results confirm the formation of a Co^I^ intermediate that can react with a proton source to form a Co^III^–H or C_2_H_2_ directly to form a Co^III^‐C_2_H_2_ adduct, suggesting that there are two possible mechanisms for acetylene reduction. We also observe a substantial decrease in evolved H_2_ from our system under 99.5% C_2_H_2_ atmosphere compared to Ar atmosphere (Figure 4e), suggesting again that H_2_ evolution and C_2_H_2_ reduction proceed via the same intermediate, and that C_2_H_2_ reduction outcompetes H_2_ evolution under our experimental conditions. It is well‐established that photocatalytic H_2_ evolution with cobaloxime catalysts proceeds via a Co^III^–H intermediate,^[^ ^18^, ^20^, ^22^ ^]^ and consequently we hypothesized that C_2_H_2_ semi‐hydrogenation also proceeds through a Co^III^–H intermediate. We observe no C_2_H_2_ reduction in the presence of the radical trap TEMPO (Figure 4f ), which also implies the existence of a radical intermediate. The formation of the TEMPO‐acetylene adduct was then confirmed by LCMS analysis of the reaction mixture (Figure 4g). We conclude that C_2_H_2_ associates with the cobaloxime catalyst through hydrogen atom transfer (HAT) as opposed to migratory insertion, as HAT generates a radical intermediate and migratory insertion does not.^[^ ^31^ ^]^ While a Co^III^–C_2_H_2_ adduct may form, we believe that this is at most a minor pathway. Since no C_2_H_4_ production is observed in the absence of a proton source, we suggest that protonation of the Co^I^ intermediate to generate a Co^III^–H is due to TFE as opposed to BIH in the full reaction mixture. While BIH can act as a proton donor once C_2_H_2_ is added, BIH is not a potent enough proton donor to react with Co^I^ directly, which explains the persistence of Co^I^ in Figure 4d prior to the addition of C_2_H_2_. In contrast, TFE can react with Co^I^ directly to form Co^III^–H, as shown in Figure 4d.

Electrochemical results of [Co(dmgH)_2_pyCl] provide further evidence of the reactivity of the Co^I^ intermediate. The cyclic voltammogram (CV) of [Co(dmgH)_2_pyCl] recorded in an Ar‐saturated solution shows a (pseudo)reversible redox peak at −1.58 V (vs Fc/Fc^+^) corresponding to the Co^II/I^ couple (Figure S15, Supporting Information). The potential of the Co^II/I^ reversible redox wave is shifted anodically by 45 mV with TFE added to the Ar‐saturated solution of [Co(dmgH)_2_pyCl], which can be attributed to the equilibrium between Co^I^ and Co^III^–H (Figure S15, Supporting Information). When the CV of [Co(dmgH)_2_pyCl] is recorded under C_2_H_2_, the addition of TFE to the electrolyte causes the rise in catalytic current coupled with the Co^II^/Co^I^ reduction event (Figure 4h). Taken together, these results offer additional support to the formation of a Co^III^–H intermediate in the photocatalytic mechanism, and that TFE is the primary source of protons in the reaction.

According to our analysis, ethylene production is initiated by Co^III^–H, followed by HAT to acetylene to form a vinyl radical that may undergo a radical pair collapse to form a vinyl‐cobalt species. Aided by density functional theory (DFT) calculations we note that the vinyl‐cobalt intermediate contains a weak Co─C bond (46.1 kcal mol^−1^), which resembles diradicals.^[^ ^17^, ^32^ ^]^ The strength of the Co^III^─H bond (49.9 kcal mol^−1^) matches the C─H bond of the vinyl radical (48.0 kcal mol^−1^), further supporting the thermodynamic feasibility of the HAT process. Calculations also support the thermodynamically favorable HAT process (Δ*G* = −17.9 kcal mol^−1^, Figure 4i; Figures S16 and S17, Supporting Information) that leads from Co─H to Co─vinyl formation. Consistently, the relatively weak Co−H (49.9 kcal mol^−1^) and Co−C (46.1 kcal mol^−1^) bond strengths in the Co─cobaloxime complexes indicate that a radical type of HAT pathway is probable.^[^ ^17^ ^]^

Overall, our proposed mechanism is as follows: chemical reduction by BIH (or photoreduction by [Ru(bpy)_2_(bpy^•−^)]^+^) reduces the Co center in [Co(dmgH)_2_pyCl] from Co^III^ to Co^II^. A subsequent photoreduction by [Ru(bpy)_2_(bpy^•−^)]^+^ yields the Co^I^ intermediate, which reacts with a proton (from TFE proton source) to form a Co^III^–H species. Then, C_2_H_2_ inserts into the Co^III^─H bond via HAT. Addition of another proton and dissociation of C_2_H_4_ regenerates Co^III^ and completes the catalytic cycle.

### 2.3 Integration of a Cobaloxime into a Metal–Organic Framework

**Figure 5:**
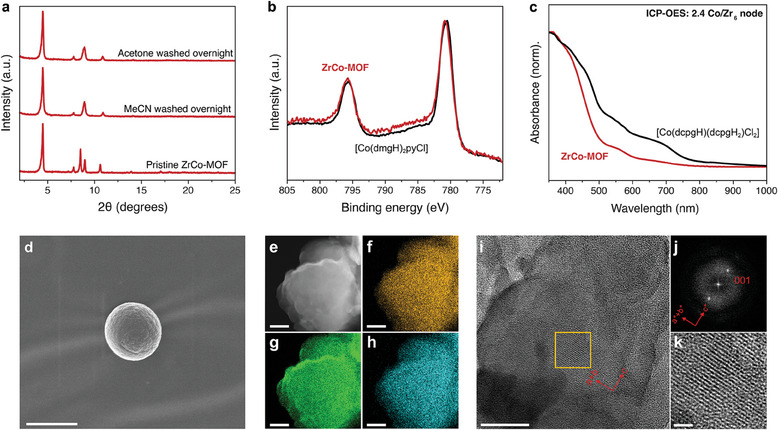
Characterization of ZrCo‐MOF. a) PXRD patterns of pristine ZrCo‐MOF, and ZrCo‐MOF soaked in acetonitrile or acetone. b) XPS spectra of [Co(dmgH)_2_pyCl] (black) and ZrCo‐MOF (red). c) DR‐UV–vis spectra of cobaloxime linker (black) and ZrCo‐MOF (red) solids. d) Scanning electron microscope image of ZrCo‐MOF after acetone wash overnight (scale bar 1 µm). e) HRTEM image of ZrCo‐MOF with corresponding energy dispersive X‐ray spectroscopy elemental maps of (f) Cl Kα, (g) Zr Lα, (h) Co Kα (scale bar 100 nm). i) HRTEM image of ZrCo‐MOF (scale bar 50 nm), j) Fourier transform of the image (i), and (k) enlarged HRTEM image from (i) (scale bar 10 nm).

To increase the robustness of our [Co(dmgH)_2_pyCl]‐based photocatalytic system and to endow recyclability, we sought to create a heterogeneous version of our photocatalytic system by using a cobaloxime as the linker in a MOF. Following previously employed strategies,^[^ ^20^, ^33^ ^]^ we synthesized a polytopic cobaloxime linker ([Co(dcpgH)(dcpgH_2_)Cl_2_], dcpgH = 4,4′‐(1,2‐bis(hydroxyimino)ethane‐1,2‐diyl)dibenzoic acid) with four carboxylate anchors for binding to hexazirconium(IV) nodes, which yielded the ZrCo‐MOF. The PXRD pattern of the obtained material shows intense peaks in the low angle range, characteristic of porous materials (**Figure** **5**a; Figure S18, Supporting Information). The two major reflections at ≈4.5° and ≈9° indicated a long‐range order along the *c*‐axis, and this is consistent with the reported partially collapsed framework structure obtained after DMF solvent removal.^[^ ^20^, ^33^ ^]^ The partially collapsed structure is also supported by SEM imaging, which shows spherical particles (Figure 5d). EDX elemental mapping of ZrCo‐MOF shows uniform distribution of cobalt and zirconium throughout the material (Figure 5e–h; Figure S19, Supporting Information). HRTEM images of ZrCo‐MOF show lattice fringes that correspond to the (001) planes (*d*_001_ = 18.3 Å) (Figure 5i–k). The ATR‐IR spectrum of the MOF shows Zr‐μ_3_‐O stretches at 640 cm^−1^ that originate from the Zr_6_ nodes (Figure S20, Supporting Information). The calculated Brunauer−Emmett−Teller surface area of ZrCo‐MOF is 827 m^2^ g^−1^ with a pore size distribution in the range of 9–15 Å (Figure S21, Supporting Information).^[^ ^20a^ ^]^ The XPS spectrum of ZrCo‐MOF is nearly identical to that of [Co(dmgH)_2_pyCl], showing that the cobalt in the MOF remains Co^III^ following MOF synthesis (Figure 5b). The UV–vis spectrum of ZrCo‐MOF indicates successful incorporation of [Co(dcpgH)(dcpgH_2_)Cl_2_] into the framework, as we observe absorption bands similar to those of the linker cobaloxime (Figure 5c). Elemental analysis using ICP‐OES reveals that there are 2.4 Co/Zr_6_ node, further supporting successful incorporation of the cobaloxime linker. ^1^H‐NMR spectroscopy of digested ZrCo‐MOF samples shows peaks at chemical shifts matching those of [Co(dcpgH)(dcpgH_2_)Cl_2_] thus confirming the structural integrity of the cobaloxime linker in the material (Figure S22, Supporting Information). The thermogravimetric analysis of ZrCo‐MOF shows a loss at ≈250 °C that can be attributed to the dehydroxylation of the Zr_6_ nodes and a loss above 400 °C of the cobaloxime linker that leads to degradation of the framework to form the metal oxide (Figure S23, Supporting Information).^[^ ^20a^ ^]^ Taken together, we conclude from these results that we successfully synthesized a Zr‐based MOF with cobaloxime linkers, thus creating a solid‐state version of our homogeneous molecular catalyst.

### 2.4 Performance of the MOF‐Based Heterogeneous System

**Figure 6:**
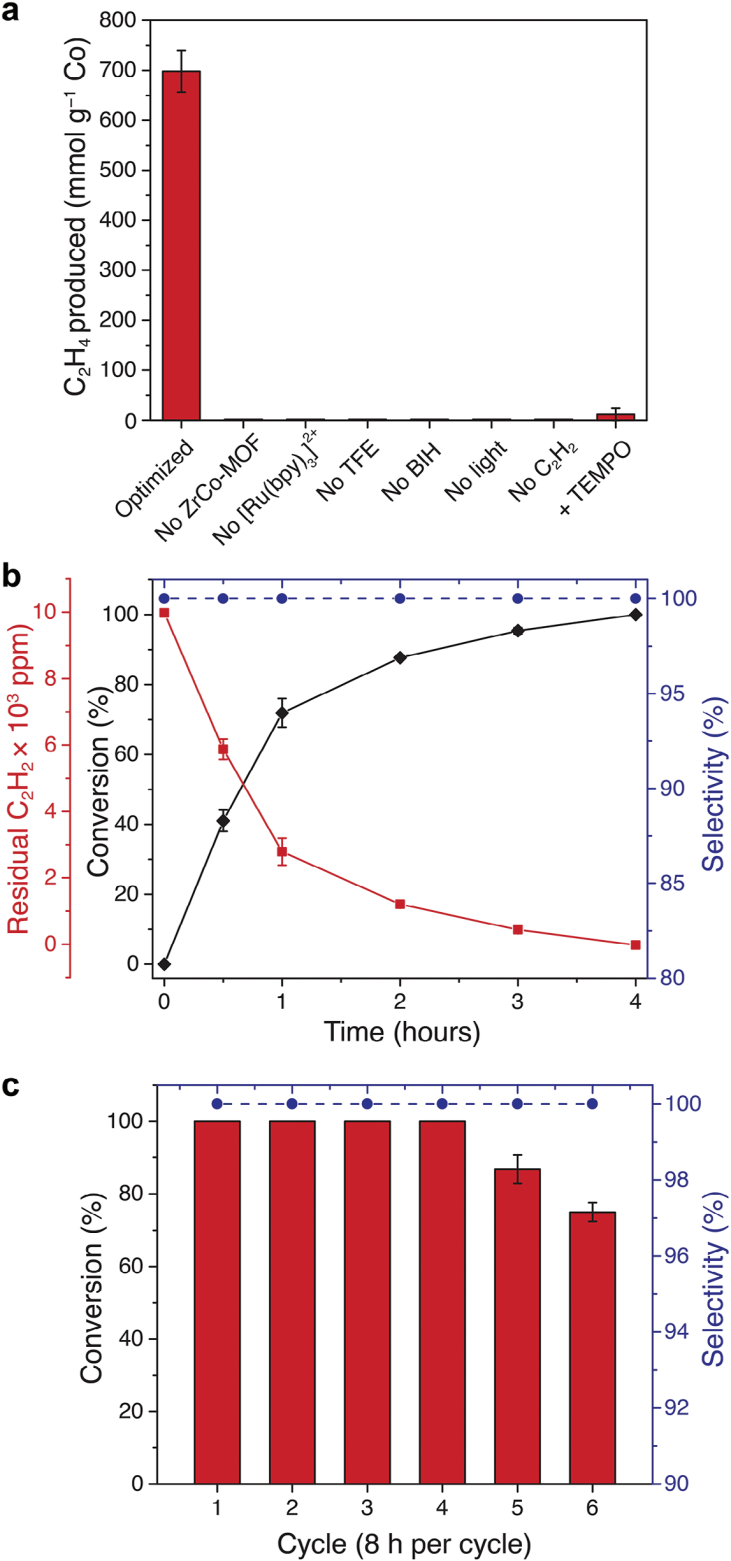
ZrCo‐MOF photocatalytic performance. a) Total C_2_H_4_ production for the optimized reaction mixture containing 2.5 mm [Ru(bpy)_3_]^2+^, 1.0 mg ZrCo‐MOF, 1.0 m TFE and 0.1 m BIH in acetonitrile irradiated (450 nm, 140 mW∙cm^−2^) for 4 h under C_2_H_2_ (≥99.5 vol.%), and for reaction mixtures that differ from the optimized conditions as indicated by the axis labels. b) Plot of C_2_H_2_ conversion (%), residual C_2_H_2_ (ppm), and C_2_H_4_ selectivity versus C_2_H_6_ (%) of the photocatalytic system containing 2.28 mg ZrCo‐MOF (1 mM cobaloxime concentration) under industrial mixture conditions (1 vol.% C_2_H_2_, 30 vol.% C_2_H_4_, He balance) as a function of irradiation time. c) Recyclability of the photocatalytic system under industrial mixture conditions (1 vol.% C_2_H_2_, 30 vol.% C_2_H_4_, He balance). Error bars represent the standard deviations for three separate experiments.

In the heterogeneous version of the photocatalytic system using ZrCo‐MOF, the catalyst achieves comparable activity and selectivity to the homogeneous system, while also displaying increased longevity, robustness, and recyclability. Incorporation of the cobaloxime catalyst as linkers of a MOF is indeed a valuable strategy to overcome the low structural stability of cobaloximes in homogeneous phase.^[^ ^18^, ^20^ ^]^ Illumination for 4 h of the ZrCo‐MOF‐based system produced 698 mmol C_2_H_4_ per gram of Co (**Figure** **6**a), which is a factor of five higher than that our previous Co‐PCN‐222‐based MOF system (142 mmol C_2_H_4_ per gram of Co).^[^ ^9^ ^]^ No C_2_H_4_ was produced in the absence of catalyst, sensitizer, sacrificial donor, proton donor, light or C_2_H_2_ feedstock (Figure 6a). Similar to the homogenous [Co(dmgH)_2_pyCl]‐based system, for the ZrCo‐MOF‐based system we observed that i) [Ru(bpy)_3_]^2+^ photoluminescence is reductively quenched in the presence of BIH (Figure S24, Supporting Information), ii) H_2_ evolution and C_2_H_2_ reduction proceed via the same intermediate, and that C_2_H_2_ reduction outcompetes H_2_ evolution under our experimental conditions (Figure S25, Supporting Information), and iii) only 11 mmol C_2_H_4_ per gram of Co are produced when we run the reaction in the presence of TEMPO (Figure 6a) that suggests the presence of a radical intermediate. Further insights into the photoreduction mechanism were obtained by using the [Co(dcpgH)(dcpgH_2_)Cl_2_] as the homogenous analogue of the ZrCo‐MOF (Figures S26 and S27, Supporting Information). Irradiation of a solution containing [Co(dcpgH)(dcpgH_2_)Cl_2_], [Ru(bpy)_3_]^2+^, and BIH results in the formation of a Co^I^ intermediate that was then consumed by addition of the TFE proton donor. Therefore, we expect that the photoreduction mechanism of acetylene to ethylene behaves in analogous manner to the homogenous cobaloxime system.

The kinetics of the ZrCo‐MOF‐based heterogeneous system under the industrially relevant conditions are shown in Figure 6b. The ZrCo‐MOF‐based system selectively photoreduces the C_2_H_2_ concentration to sub‐ppm levels after 4 h of illumination. We prepared the samples of the MOF system to have the same number of moles of cobaloxime as the homogeneous samples; we speculate that the MOF‐based system takes longer to achieve complete C_2_H_2_ conversion due to diffusional limitations and reduced access to the catalytic sites on the interior of the framework. We note, however, that this is still a drastic improvement compared to the 87 h required for our previous Co‐PCN‐222‐based MOF system^[^ ^9^ ^]^ to fully remove C_2_H_2_ from the industrial mixture, and compared to the 68 h required by recently reported metal–organic framework nanosheets^[^ ^34^ ^]^ to reduce acetylene concentration below 5 ppm. Crucially, like the homogeneous system, no over‐hydrogenation to C_2_H_6_ is observed, such that the selectivity for C_2_H_4_ remains >99.9% (Figure 6b). Sorption isotherms demonstrate a higher Henry's adsorption constant for acetylene than that for ethylene ($K_{H}^{C_{2}H_{2}}$ = 3.17 × 10^−3^ mmol g^−1^ torr^−1^ and $K_{H}^{C_{2}H_{4}}$ = 2.29 × 10^−3^ mmol g^−1^ torr^−1^ as determined from the linear region of each isotherm, Figure S28, Supporting Information). We expect that, however, the propensity of ZrCo‐MOF to selectively adsorb either C_2_H_2_ or C_2_H_4_ will be very different in the solution compared to the gas phase. Indeed, we exclude C_2_H_2_ adsorption effects on the photoreduction reaction by the ZrCo‐MOF‐based heterogeneous system. This conclusion is corroborated by the unchanged C_2_H_2_ content that is measured for the ZrCo‐MOF‐based heterogeneous system under C_2_H_4_/C_2_H_2_ either before the 4 h of illumination or after being kept in the dark for 4 h (Figure S29, Supporting Information).

The homogeneous system persists for three cycles and 4 h in total before losing its ability to fully reduce C_2_H_2_ in an industrially relevant mixture. In contrast, ZrCo‐MOF can be recycled up to six times, and remains catalytically active for 48 h before significant loss of performance (Figure 6c). PXRD patterns collected periodically over the course of the reaction demonstrate retention of crystallinity, even after 48 h of irradiation (Figure S30, Supporting Information). Similarly, SEM images taken at various time points indicate retention of particle morphology (Figure S31, Supporting Information). Furthermore, elemental analysis of the MOF powder after photocatalysis indicates that only a small amount of Co leaching occurs over the course of the reaction: according to ICP‐OES, there are 2.3 Co/Zr_6_ node after both 4 and 24 h of continuous photocatalysis, and 2.0 Co/Zr_6_ node after 48 h of photocatalysis, inclusive of repeated centrifugation and washing of the recycling experiment (Table S1, Supporting Information). Compared with the 2.4 Co/Zr_6_ node present in pristine ZrCo‐MOF, this represents a small loss of Co, and we therefore conclude that the loss of catalytic activity is not due to Co leaching, which is further corroborated by ICP‐OES of the supernatant from the catalytic mixture after 24 h of catalysis indicating that only 10.4 ± 1.6% of cobalt in the original ZrCo‐MOF leaches in that time. We therefore speculate that the reason catalysis ceases is due to pore clogging with other reaction components over time.

## 3 Conclusion

In conclusion, we have developed a novel photocatalytic system based on a molecular cobaloxime catalyst for the semi‐hydrogenation of acetylene to ethylene, under visible light irradiation and at room temperature. In homogenous conditions, at dilute catalyst conditions, the system achieves TON >1000 after 20 h of illumination with >99.9% selectivity for ethylene versus ethane. At concentrated catalyst conditions, the system produces 63 µmol C_2_H_4_ after 4 h of irradiation with a quantum yield (QY) of 1.0%, a 10‐ and 50‐fold improvement over our recently investigated Co‐porphyrin or Co‐PCN‐222 system, respectively.^[^ ^8^, ^9^ ^]^ Furthermore, the cobaloxime‐based system completely reduces the acetylene in a dilute acetylene co‐feed with ethylene (the industrially relevant conditions) without any detectable over‐hydrogenation to ethane in just 50 min, which represents a more than 30‐ and 100‐fold improvement over our recently investigated Co‐porphyrin or Co‐PCN‐222 systems, respectively.^[^ ^8^, ^9^ ^]^ The production efficiency of our system is ≈13 L g^−1^ h^−1^, which exceeds the industrial demand of >10 L g^−1^ h^−1^. We note, however, that comparing the present system with other thermo‐ and electro‐catalytic routes is challenging due to dissimilar conditions (amount and state of catalysts, proton sources, etc.) and that the present system still requires the use of sacrificial electron donors, which ultimately increases its cost.

Investigation of the mechanism reveals the importance of a Co^I^ species that becomes protonated to form Co^III^–H (i.e., Co^III^–hydride), to which acetylene inserts via HAT. To improve the durability and recyclability of the homogeneous system, we engineered a heterogeneous version by using a molecular cobaloxime as the linker in a MOF. With the industrial mixture composition, our MOF‐based system exhibited the same excellent selectivity (albeit with slightly lower activity) as the homogeneous system, reducing the acetylene concentration to sub‐ppm levels with no observed over‐hydrogenation within 4 h, which represents a ≈20‐fold improvement over the two recently reported MOF‐based systems.^[^ ^8^, ^9^ ^]^ Importantly, our MOF‐based system displayed increased longevity and recyclability over the homogeneous system, maintaining catalytic activity for 48 h over six cycles.

Besides the higher activity of both the homogeneous and heterogeneous systems compared to their Co‐porphyrin counterparts, ZrCo‐MOF exhibits greater stability under the reaction conditions than Co‐PCN‐222,^[^ ^9^ ^]^ most likely due to the elimination of triethanolamine as sacrificial donor. A further improvement to the system described here would be the elimination of organic solvent: while we demonstrate that the [Co(dmgH)_2_pyCl]‐based system can use water as the source of protons, ZrCo‐MOF lacks the requisite stability in water to make such a heterogeneous system feasible. Alternative MOF‐based systems based on water‐stable MOFs should be investigated. While cobaloximes are cheaper and easier to prepare than porphyrins, the preparation of [Co(dcpgH)(dcpgH_2_)Cl_2_] linker is a multi‐step synthesis and therefore simpler cobalt‐containing MOFs, such as MOFs that utilize Co as the node^[^ ^35^ ^]^ or incorporation of catalysts through post‐synthetic incorporation,^[^ ^14^, ^36^ ^]^ is an intriguing direction with more potential for industrial scale up. In the future, the use of sacrificial donors could be avoided by coupling the oxidative reaction to produce value‐added products. Another improvement to our system would be to exploit this catalytic cycle in electrochemical thin films^[^ ^20^ ^]^ or a photoelectrochemical cell, thus avoiding altogether the use of the sacrificial electron donor. Another exciting possibility is to combine the adsorption capacity for acetylene of previously reported porous materials^[^ ^37^ ^]^ with the (photo)catalytic properties reported here.

Overall, the photocatalytic strategies described here avoid the use of noble metal catalysts, high temperatures and pressures, and H_2_ gas feed, all while achieving significantly higher selectivity than the current industry standard. This work is a significant step forward for the photocatalytic production of polymer‐grade ethylene in molecular and porous catalysts. We anticipate that this work will stimulate further exploration into photo‐ and electro‐catalytic activity^[^ ^38^ ^]^ of cobaloximes and MOF‐based systems for the semi‐hydrogenation of acetylene to ethylene and their integration into flow cells for crude ethylene purification, as well as motivate its use for other selective hydrogenation reactions and other alkyne substrates.

## Conflict of Interest

The authors declare no conflict of interest.

## Author Contributions

A.E.B.S.S. and A.F. contributed equally. L.Ð., F.A., and J.T.H. proposed and supervised the project. A.E.B.S.S., L.Ð., and F.A. designed the experiments. A.E.B.S.S., L.Ð., F.A., and A.F. carried out the experiments, with help E.S. X.W., L.Ð., and R.Q.S. designed, performed and analyzed first‐principles calculations. L.Ð., A.E.B.S.S., F.A., and J.T.H. wrote the manuscript. All authors were involved in manuscript revision and have approved the final version of the manuscript.

## Supporting information



Supporting Information

## Data Availability

The data that support the findings of this study are available from the corresponding authors upon request.
