# Supplementary material for: Photocatalytic Semi‐Hydrogenation of Acetylene to Polymer‐Grade Ethylene with Molecular and Metal–Organic Framework Cobaloximes
Source: Adv Mater. 2024 Oct 22;37(1):2408658. doi: 10.1002/adma.202408658 (PMC11707558; doi:10.1002/adma.202408658)
Supplement: Supplementary file 1 — Supporting Information [file ADMA-37-2408658-s001.pdf]

ADVANCED MATERIALS

Supporting Information

for *Adv. Mater.*, DOI 10.1002/adma.202408658

Photocatalytic Semi-Hydrogenation of Acetylene to Polymer-Grade Ethylene with Molecular and Metal–Organic Framework Cobaloximes

Aaron E.B.S. Stone, Anna Fortunato, Xijun Wang, Edoardo Saggioro, Randall Q. Snurr, Joseph T. Hupp, Francesca Arcudi* and Luka Đorđević**

Supplementary Information for

Photocatalytic Semi-Hydrogenation of Acetylene to Polymer-Grade Ethylene with Molecular and Metal-Organic Framework Cobaloximes

Aaron E.B.S. Stone,[1,†] Anna Fortunato,[2,†] Xijun Wang,[3] Edoardo Saggioro,[2] Randall Q. Snurr,[3] Joseph T. Hupp,[1,*] Francesca Arcudi,[2,*] and Luka Đorđević[2,*]

[1]*Department of Chemistry, Northwestern University, 2145 Sheridan Rd., Evanston, IL 60208-3113, United States*
[2]*Department of Chemical Sciences, University of Padova, Via F. Marzolo 1, 35131 Padova, Italy*
[3]*Department of Chemical and Biological Engineering, Northwestern University, 2145 Sheridan Rd., Evanston, IL 60208-3120, United States*

[†]These authors contributed equally.
*corresponding authors. Emails: luka.dordevic@unipd.it; francesca.arcudi@unipd.it; j-hupp@northwestern.edu;

Table of Contents

Materials and Methods 3

- Safety warning 3
- Materials 3
- Photocatalytic reactions 7
- Chromatographic detection of gases 8
- Chromatographic detection of liquids 11
- Quantum yield 11
- Absorption spectroscopy 12
- Fluorescence quenching 12
- Electron paramagnetic spectroscopy 14
- Electrochemical characterization 14
- Detection of the radical intermediate 15
- Powder X-ray diffraction 15
- Elemental analysis 15
- Scanning electron microscopy 16
- Transmission electron microscopy 16
- Infrared Spectroscopy 17
- Thermogravimetric analysis 17
- X-ray photoelectron spectroscopy 17
- Gas sorption isotherms 17
- First-principles calculations 18

Supplementary Figures 20

Supplementary Tables 49

Supplementary References 50

Materials and Methods

Safety warning

Acetylene is an extremely flammable gas. Buildup of acetylene vapors can result in fire or explosions if triggered by sparks. Acetylene may displace oxygen and cause rapid suffocation. In our experimental setup, the acetylene cylinder was fitted with a CGA 510 regulator equipped with a flashback arrestor and connected, through stainless steel tubing and a flow regulator, to a purging station that was placed inside a fume hood.[1] A Snoop® solution was applied to fittings and joints to inspect for leaks, until no bubble formation was observed. There was no electrical equipment in the fume hood.

Materials

Chloro(pyridine)bis(dimethylglyoximato)cobalt(III) ($Co(dmgH)_2pyCl$, Sigma-Aldrich, 341630-5G, batch 0000208292), tris(2,2′-bipyridyl)dichlororuthenium(II) hexahydrate ($Ru(bpy)_3^{2+}$, Sigma-Aldrich, 99.95%), phenylacetylene (Sigma-Aldrich, 98%), styrene (Sigma-Aldrich), 2,2,2-trifluoroethanol (TFE, Sigma-Aldrich, ≥99%), 2,2,2-Trifluoroethanol-d_3 (Sigma-Aldrich, ≥99.5 atom % D), 1,3-dimethyl-2-phenyl-2,3-dihydro-1*H*-benzo[*d*]imidazole (BIH, BLDpharm, 97%), phenol (Sigma-Aldrich), 1,1,1,3,3,3-hexafluoro-2-propanol (HFIP, Sigma-Aldrich, ≥99%), (2,2,6,6-tetramethylpiperidin-1-yl)oxyl (TEMPO, 99%, Sigma-Aldrich), acetonitrile (ACN, Sigma-Aldrich, anhydrous 99.8%), triethanolamine (TEOA, Sigma-Aldrich, >99.0%,) were used as received. Ultrapure water (>18.25 MΩ cm) was obtained using a Thermo Scientific Barnstead GenPure Pro.

Synthesis of cobaloximes and ZrCo-MOF

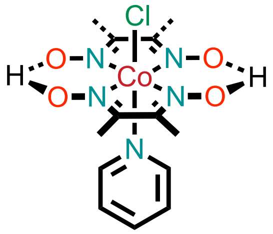

[Co(dmgH)$_2$pyCl]

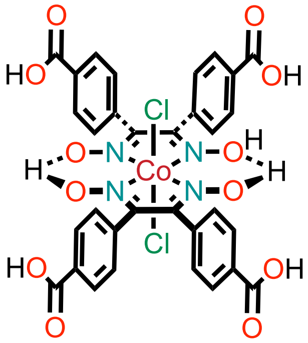

[Co(dcpgH)(dcpgH$_2$)Cl$_2$]

Chloro(pyridine)cobaloxime [Co(dmgH)$_2$pyCl] was synthesized according to a modified literature procedure.[2] To a hot solution (50 °C) of cobalt(II) chloride hexahydrate (2.0 g, 8.4 mmol) and dimethylglyoxime (2.1 g, 18.4 mmol) in absolute ethanol (80 mL), was added pyridine (1.4 mL, 17.2 mmol). The mixture was left to cool down to room temperature. A stream of air was passed through the mixture via Pasteur pipette for 30 minutes. The reaction mixture was then left standing for 1 h at room temperature. The solid was collected by filtration and the solid was washed with water (20 mL), ethanol (20 mL), diethyl ether (20 mL), and was left to dry at room temperature. The product was obtained as brown solid (2.4 g, 71% yield). *Anal*. Calcd. dor $C_{18}H_{19}N_5O_4ClCo$: C, 38.67%; H, 4.74%; N, 17.35%. Found 38.41%; H, 4.49%; N, 17.03%.

The elemental analysis reported for the commercial [Co(dmgH)$_2$pyCl], obtained and used as received from Sigma-Aldrich (341630-5G, batch 0000208292), is C 39.6% and N 17.6%.

4,4′-(1,2-bis(hydroxyimino)ethane-1,2-diyl)dibenzoate hydroxylammonium salt, the corresponding cobaloxime [Co(dcpgH)(dcpgH$_2$)Cl$_2$], (dcpgH = diphenylglyoxime-4,4’-dicarboxylic acid) and subsequently ZrCo-MOF were synthesized according to published

procedures.[3] Note: we found that $[Co(dcpgH)(dcpgH_2)]Cl_2$ could be prepared only when using the hydroxylamonium salt of 4,4′-(1,2-bis(hydroxyimino)ethane-1,2-diyl)dibenzoate, while when using 4,4′-(1,2-bis(hydroxyimino)ethane-1,2-diyl)dibenzoic acid,[4] the cobaloxime formation was not observed.

Synthesis of 1,3-dimethyl-2-phenyl-2,3-dihydro-1H-benzo[d]imidazole (BIH)

When performing experiments with commercial BIH (BLDpharm, BD742561 97%) we observed a lower solubility in organic solvents compared to BIH prepared by us. We also noticed lower photocatalytic performances when using commercial BIH. We prepared BIH according to slightly modified literature procedures,[5] as detailed below.

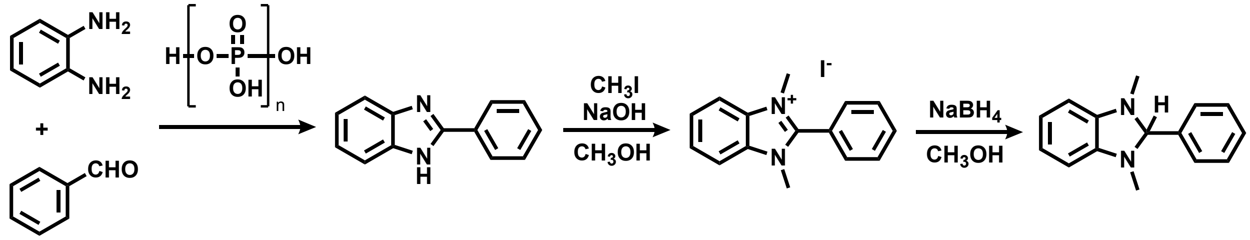

2-Phenyl-1*H*-benzo[*d*]imidazole: Benzoic acid (12.3 g, 100.4 mmol), *o*-phenylenediamine (10.8 g, 100.4 mmol) and polyphosphoric acid (40.0 g) were combined in a round-bottom flask and heated to 180 °C for 2 hours. The reaction mixture was left to cool down, and 2 M aq. NaOH was added until the pH was neutral. The off-white solid was filtered, washed with water, and finally dried at 60 °C under vacuum (15.5 g, 95% yield). ^{1}H NMR (400 MHz, DMSO-d_6) δ 8.18 (d, J = 7.2 Hz, 2H), 7.67 (d, J = 7.6 Hz, 1H), 7.60 – 7.40 (m, 4H), 7.27 – 7.12 (m, 2H).

1,3-dimethyl-2-phenyl-1*H*-benzo[*d*]imidazole-3-ium iodide: To a mixture of 2-phenyl-1*H*-benzo[*d*]imidazole (15.0 g, 77.2 mmol) in methanol (100 mL) was first added NaOH (3.21 g, 80.3 mmol), followed by methyl iodide (17.5 mL, 282 mmol). The mixture was refluxed for 24 hours. At this point, additional NaOH (0.88 g) and CH_3I (4.8 mL) were added, and the mixture was refluxed for 18 hours. Then, the volatiles were removed under reduced pressure, a mixture of ethanol/water (200 mL, 5:1 *v*/*v*) and active carbon were added, and the mixture was stirred at 80 °C for 1 hour. The hot mixture was filtered over celite, and the filtrate was concentrated under reduced pressure. The solid was purified by crystallization (from boiling ethanol/water, 200 mL, 5:1 *v*/*v*). The mixture was filtered, washed with water and cold ethanol, and dried under vacuum to obtain the product as white needle crystals (20.3 g, 75% yield). ^{1}H NMR (400 MHz, DMSO-d_6) δ 8.17 – 8.10 (m, 2H), 7.93 – 7.88 (m, 2H), 7.87 – 7.74 (m, 5H), 3.90 (s, 6H).

1,3-dimethyl-2-phenyl-2,3-dihydro-1*H*-benzo[*d*]imidazole (BIH): To a mixture of 1,3-dimethyl-2-phenyl-1*H*-benzo[*d*]imidazole-3-ium iodide (17.0 g, 48.5 mmol) in dry methanol (0.48 L) was added portion wise $NaBH_4$ (5.50 g, 145 mmol) at 0 °C. After the addition was finished, the mixture was left to warm up to room temperature and left stirring for 1 hour. The solvent was removed under reduced pressure and the product was obtained after crystallization from hot ethanol/water (2:1 *v*/*v*). The crystals were recovered by filtration, washed with water and cold ethanol, and finally dried. The filtrate was concentrated under reduced pressure and re-crystallized as detailed above. The crystals were combined (10.0 g, 92% yield). ^{1}H NMR (400 MHz, DMSO-d_6) δ 7.57 – 7.52 (m, 2H), 7.47 – 7.42 (m, 3H), 6.62 (dd, J = 5.5, 3.2 Hz, 2H), 6.45 (dd, J = 5.4, 3.2 Hz, 2H), 4.87 (s, 1H), 2.48 (s, 6H).

Photocatalytic reactions

Samples were prepared in a 9.0 mL screw cap vial (B7800-3, Thermo Scientific) equipped with a micro stir bar (7 mm, Fisher Scientific) and sealed with silicone/PTFE septum (TS-12713, Thermo Scientific) and cap (open top, TS-13216, Thermo Scientific).

Vials for the semi-hydrogenation of acetylene were sealed and purged, through a flow regulator (at 130 mm using a Key Instruments 150 mm glass tube flowmeter), either for (*i*) 10 minutes with He (or Ar) followed by 5 minutes with C_2H_2 (≥99.5 vol.%, Airgas) or (*ii*) for 15 minutes with C_2H_4/C_2H_2 mixture (1 vol.% C_2H_2, 30 vol.% C_2H_4, He balance, Airgas) or (*iii*) 15 minutes with Ar by using steel needles inserted through the septum as inlet (inserted into the solution) and outlet (venting the headspace to the surrounding atmosphere).

Vials for the semi-hydrogenation of phenylacetylene were charged with [Co(dmgH)$_2$pyCl] (10 mol%), $[Ru(bpy)_3]^{2+}$ (2.5 mM), BIH (0.1 M) and phenylacetylene (10 mM). To the mixture, acetonitrile (2.0 mL) and TFE (1.0 M) were added, and then the vials were sealed and purged, through a flow regulator (at 130 mm) for 15 minutes with Ar by using steel needles inserted through the septum as inlet (inserted into the solution) and outlet (venting the headspace to the surrounding atmosphere).

After purging for the time specified above, the pressure of the headspace was then equilibrated to 1 atm. The vials were then illuminated using a homebuilt photoreactor made of royal blue (450 nm) LEDs (Cree XLamp XP-E2 Color High Power LED Star, LEDsupply.com) with a light intensity of 140 $mW\cdot cm^{-2}$ (measured using an Optical Power Meter PM100D with Optical Sensor S120VC from Thorlabs). Each vial was suspended on top of a single LED, equipped with a lens,

using a homebuilt sample holder. The vials were continuously stirred at 600 rpm during the irradiation.

The sample of Figure 3c was a 2.0 mL C_2H_2/C_2H_4 (1 vol.% C_2H_2, 30 vol.% C_2H_4, He balance)-purged solution in acetonitrile containing 2.5 mM $[Ru(bpy)_3]^{2+}$, 1.0 mM $[Co(dmgH)_2pyCl]$, 1.0 M TFE and 0.1 M BIH. Following irradiation for 1 h (450 nm, 140 $mW\cdot cm^{-2}$), the headspace was analyzed with GC-FID (as reported below) to ensure reduction of C_2H_2 to undetectable levels. For cycles 2-4, the sample was purged with C_2H_2/C_2H_4 (1 vol.% C_2H_2, 30 vol.% C_2H_4, He balance) upon addition of fresh 2.5 mM $[Ru(bpy)_3]^{2+}$, 1.0 M TFE and 0.1 M BIH.

The sample of Figure 6c was a 2.0 mL C_2H_2/C_2H_4 (1 vol.% C_2H_2, 30 vol.% C_2H_4, He balance)-purged solution in acetonitrile containing 2.5 mM $[Ru(bpy)_3]^{2+}$, 2.28 mg ZrCo-MOF, 1.0 M TFE and 0.1 M BIH. Following irradiation for 4 h (450 nm, 140 $mW\cdot cm^{-2}$), the headspace was analyzed with GC-FID (as reported below) to ensure reduction of C_2H_2 to undetectable levels. For cycles 2-6, the used catalyst is recovered and redispersed in a fresh solution containing 2.5 mM $[Ru(bpy)_3]^{2+}$, 1.0 M TFE and 0.1 M BIH, and the sample was then purged with C_2H_2/C_2H_4 (1 vol.% C_2H_2, 30 vol.% C_2H_4, He balance).

Chromatographic detection of gases

Gas chromatograms and mass spectra were collected on an Agilent Technologies 6850 Network GC system coupled with a 5975C VL MSD with Triple-Axis Detector. The GC was equipped with a HP-PLOT Q column, the inlet temperature was 250 °C, the He carrier gas flow was 1.2 $mL\cdot min^{-1}$ at a pressure of 4.30 psi. For the detection of ethylene, acetylene, and ethane the oven temperature was kept at 45 °C for 4.50 min, and then heated to 200 °C using a 30 $°C\cdot min^{-1}$ ramp (total run time

9.67 min). Headspace samples were manually injected using gas-tight Hamilton sample-lock syringes (100-250 μL). The calibration curve for C_2H_4 was collected by injecting known quantities of a gas mixture containing C_2H_4 (2 vol.% standard, He balance, Airgas). The calibration curve for C_2H_6 was collected by injecting known quantities of a gas standard containing C_2H_6 (100 and 4200 ppm). The selectivity for C_2H_4 is reported as >99.9% when no quantifiable C_2H_6 and H_2 are detected in the gas chromatograms.

Turnover number (TON) of C_2H_4 produced, amount of C_2H_4 produced per gram catalyst (mmol·g^{-1} Co), and selectivity for ethylene vs. C_2H_6 and H_2 ($S_{C_2H_4}^{total}$) and vs. just C_2H_6 ($S_{C_2H_4}^{C_2H_6}$) for the photoreduction of C_2H_2 (≥99.5 vol.%, Airgas) were calculated as follows:

$$\mathrm{TON}_{C_2H_4} = \frac{(\mathrm{mol}\ C_2H_4)}{(\mathrm{mol}\ [\mathrm{Co(dmgH)_2)pyCl}])}$$

$$\mathrm{Amount}_{C_2H_4}\ (\mathrm{mmol\cdot g^{-1}\ Co}) = \frac{(\mathrm{mol}\ C_2H_4)}{(\mathrm{g\ ZrCo-MOF})(\mathrm{wt.\%\ Co\ in\ ZrCo-MOF})}$$

$$S_{C_2H_4}^{total}\ (\%) = \frac{\mathrm{mol}\ C_2H_4}{\mathrm{mol}\ C_2H_4 + \mathrm{mol}\ C_2H_6 + \mathrm{mol}\ H_2} \times 100$$

$$S_{C_2H_4}^{C_2H_6}\ (\%) = \frac{\mathrm{mol}\ C_2H_4}{\mathrm{mol}\ C_2H_4 + \mathrm{mol}\ C_2H_6} \times 100$$

Experiments were performed at least in duplicate.

For the detection and quantification of acetylene and ethane in the photoreduction of the C_2H_4/C_2H_2 mixture (1 vol.% C_2H_2, 30 vol.% C_2H_4, He balance, Airgas) a custom-built Shimadzu GC-2014 gas chromatography system equipped with flame ionization detector (FID) was used. The column used was HayeSep T (1/16", 7.5 m) with an argon carrier gas flow of 7.5 mL·min^{-1} min at constant pressure of 2.5-2.7 bars, and the FID detector maintained at 250 °C. The oven

temperature was kept at 35 °C for 9.0 min, and then heated to 85 °C using a 40 °C·min^{-1} ramp, and kept at 85 °C for 4.0 min (total run time 17.0 min). Calibration curves for C_2H_2 and C_2H_6 were collected by injecting known quantities of a standard gas mixture (TOGAS 4200 ppm standard and 1% C_2H_2 in C_2H_4 and He). Experiments were performed at least in duplicate. The intercept of the calibration curves for C_2H_2 and C_2H_6 crosses zero to guarantee accuracy when the gas concentration is as low as few ppm. Injections were performed using gas-tight Hamilton syringes (10-25 μL). C_2H_2 conversion ($C_{C_2H_2}$) and selectivity for ethylene (S_{C2H4}) for the photoreduction of the C_2H_4/C_2H_2 mixture were calculated as follows:

$$C_{C_2H_2}\,(\%) = \frac{[C_2H_2]_{feed} - [C_2H_2]_x}{[C_2H_2]_{feed}} \times 100$$

$$S_{C_2H_4}\,(\%) = \frac{[C_2H_2]_{feed} - [C_2H_2]_x}{[C_2H_2]_{feed} - [C_2H_2]_x + [C_2H_6]} \times 100$$

where $[C_2H_2]_{feed}$ represents the acetylene concentration in the feed (1×10^4 ppm) and $[C_2H_2]_x$ and $[C_2H_6]$ are the concentrations of acetylene and ethane in the product. The change in ethylene concentration cannot be accurately measured because of the excess of ethylene in the gas feed and therefore is not used for calculating the selectivity.[6] The production efficiency was calculated using 9 mL volume reactor, the mass of the catalyst and the reaction time.[7]

Analyses of H_2 gas evolved in the headspace during the photocatalysis were performed with a custom-built Shimadzu GC-2014 gas chromatography system equipped with a thermal conductivity detector. H_2 production was quantitatively detected using HayeSep T (1/16", 7.5 m) and MS-5A (1/16", 2.5 m) columns. The temperature was held at 100 °C for the TCD detector and 40 °C for the oven. The carrier gas was argon flowing at 8.5 mL·min^{-1}, at constant pressure of 3.8-4.0 bars. Injections (100 μL) were performed *via* an autosampler (AOC 6000) equipped with a gas-

tight syringe (SGE autosampler syringe). Calibration curve for H_2 was collected by injecting known quantities of H_2 (5 vol.%, standard, Ar balance, Airgas). Experiments were performed at least in duplicate.

Chromatographic detection of liquids

GC-MS experiments were performed on an Agilent Technologies 6850 Network GC system coupled with a 5975 Mass Selective Detector. The GC was equipped with a HP-5ms column, the inlet temperature was 220 °C, the He carrier gas flow was 1.0 mL·min^{-1} at a pressure of 7.7 psi.

Quantum yield

The quantum yield of a photochemical process is calculated as the number of defined events occurring per photon absorbed by the system at a specific wavelength. The $\Phi_{C_2H_4}(\%)$ was therefore calculated according to the following equation:

$$\Phi_{C_2H_4}\,(\%) = \frac{\text{number of } C_2H_4 \text{ molecules } \times 2}{\text{number of photons absorbed}} \times 100$$

To calculate the fraction of photons absorbed, we determined the amount of absorbed light at the beginning of the photocatalytic experiments from (at least) three independent readings of the measured power at the top of the reaction vessel (an Optical Power Meter PM100D with Optical Sensor S120VC from Thorlabs was used). The reaction vessel contained a 2.0 mL solution of BIH (0.1 M) and TFE (1.0 M) to account for the reflection loss at the glass/air interface. The number of photons absorbed was calculated taking the photon wavelength equal to 450 nm, an incident

light power of 140 mW·cm^{-2} and considering an illuminated area of 1.767 cm^2. Under our conditions, 2.0 mL of a solution containing 2.5 mM $[Ru(bpy)_3]^{2+}$, 1 mM $[Co(dmgH)_2pyCl]$, 1.0 M TFE and 0.1 M BIH absorbed 96% of incident photons. The number of molecules of C_2H_4 were determined from the moles of C_2H_4 in the sample headspace (obtained by GC measurements) from three independent experiments (at 4 h of irradiation). The $\Phi_{C_2H_4}$ was calculated to be 1.0%.

Absorption spectroscopy

UV–vis absorption spectra were recorded on a Varian Cary 5000 spectrometer. UV-vis spectra of MOF powders were collected using an Agilent Technologies Internal DRA 2500 diffuse-reflectance UV-vis attachment. We prepared ~1 mg/mL suspensions of each MOF in acetone and drop-cast 100-300 μL onto Platinum Line® Cover Glass microscope coverslips (22 x 22 #1.5). Sealable quartz cuvettes (Starna Cells with septum cap) were used for investigation of $[Co(dmgH)_2pyCl]$, equipped with a micro stir bar and the solutions were prepared and degassed as described for the photocatalytic systems. Irradiation was performed with a 450 nm LED (140 mW·cm^{-2}). Acetonitrile purged with He and/or C_2H_2 was used as baseline.

Fluorescence quenching

For the fluorescence quenching experiments, solutions of 50 μM $[Ru(bpy)_3]^{2+}$ in acetonitrile containing various concentrations of $[Co(dmgH)_2pyCl]$ or BIH were purged for 10 minutes with He followed by 5 minutes with C_2H_2 (≥99.5 vol.%). The lifetimes were measured in a custom-built microscope equipped with a piezo scanner (NanoPI, Physik Instrumente), an APD detector (MicroPhoton Devices), and a photon counting board (PicoHarp300, PicoQuant) where correlation

times between the excitation pulses and detected photons were recorded. The excitation pulses were synchronized from a 440 nm, 70 ps pulsed diode laser at a repetition rate of 250 kHz (Picoquant) and focused with a long working distance objective (0.7 NA, 100×, Mitutoyo), and the detected fluorescence was filtered with a 490 nm long-pass dichroic (Thorlabs) and a 495 nm long-pass filter (Thorlabs). The quenching rate constant (k_q) was calculated according to the Stern-Volmer equation:

$$\frac{\tau_0}{\tau} = 1 + k_q \times \tau_0 \times [Q]$$

where τ_0 and τ are the lifetime of $[Ru(bpy)_3]^{2+}$ in absence (τ_0 = 957 ns) and in presence of quencher and $[Q]$ is the molar concentration of the quencher.

Figure 4c: $[[Co(dmgH)_2pyCl]]$ = 0-1.25 mM, [BIH] = 0-100 μM. The measured bimolecular rate constants are $k_q^{cobaloxime} = 6.79 \times 10^9$ $M^{-1}\cdot s^{-1}$ and $k_q^{BIH} = 6.15 \times 10^9$ $M^{-1}\cdot s^{-1}$, which correspond to unimolecular rate constants k_q[cobaloxime] = 6.79×10^6 s^{-1} and k_q[BIH] = 6.15×10^8 s^{-1} for the concentrations of these species ($[[Co(dmgH)_2pyCl]]$ = 1.0 mM, [BIH] = 0.1 M) used in our catalytic reaction mixtures.

Figure S24: Linker $[[Co(dcpgH)(dcpgH_2)Cl_2]]$ = 0-84.4 μM, [BIH] = 0-100 μM. The measured bimolecular rate constants are $k_q^{Linker} = 1.10 \times 10^{10}$ $M^{-1}\cdot s^{-1}$ and $k_q^{BIH} = 4.8 \times 10^9$ $M^{-1}\cdot s^{-1}$, which correspond to unimolecular rate constants k_q[Linker] = 4.82×10^6 s^{-1} and k_q[BIH] = 4.8×10^8 s^{-1} for the concentrations of these species ($[[Co(dcpgH)(dcpgH_2)Cl_2]]$ = 0.44 mM, [BIH] = 0.1 M) used in our catalytic reaction mixtures (1 mg ZrCo-MOF = 0.44 mM cobaloxime linker concentration).

Electron paramagnetic spectroscopy

Electron Paramagnetic resonance (EPR) spectra were acquired on a Bruker ESR5000 operating at a microwave frequency of 9.46 GHz. In a dry box, a deoxygenated solution of [Co(dmgH)$_2$pyCl] (1 mM) and BIH (10 mM) was prepared, then 250 µL of this solution was immediately transferred to an EPR tube (4 mm) that was sealed under inert atmosphere. The sample was then transferred out of the dry box and frozen in liquid N_2. The EPR spectra were recorded using ESRStudio at 77 K with 5.0 mT microwave power, 1.0 mT modulation and 120 seconds sweep time, and finally baseline-corrected.

Electrochemical characterization

Cyclic voltammetry (CV) was performed on a Pine Research WaveDriver 100 potentiostat at room temperature, employing a standard three-electrode single-compartment cell: glassy carbon electrode (GCE, CH Instruments, d = 3 mm) as working electrode, a Pt wire as counter electrode and Ag wire as pseudoreference electrode. Working and reference electrodes were polished on a felt pad with 0.3 or 0.05 µm Al_2O_3 suspensions, sonicated in deionized water for about 30 seconds and washed/dried before each experiment; the Pt wire was flame-cleaned. A blank scan was recorded before each sample (scan rate = 50 $mV{\cdot}s^{-1}$). Cobaloxime solution (5 mM) was prepared in spectroscopic grade acetonitrile, using TBA•PF_6 (0.1 M) as supporting electrolyte and purged for 10 minutes either with argon or acetonitrile before recording the CV.

Detection of the radical intermediate

Radical intermediate was detected by TEMPO trapping. Samples for LCMS analysis were prepared identically to standard photocatalysis samples, except with the addition of 200 molar equiv. 2,2,6,6-tetramethylpiperidin-1-oxyl (TEMPO) relative to [Co(dmgH)$_2$pyCl]. Samples were irradiated for 4 h with 450 nm light (140 mW·cm^{-2}). LCMS samples were then prepared by diluting the neat reaction mixture using HPLC grade acetonitrile. LCMS samples were analyzed using a high-resolution mass spectrometry coupled to a reversed-phase chromatography system (Agilent 6545 Q-TOF LC/MS). Using the Agilent Qualitative Analysis "Find By Formula" workflow, the molecular formula of $C_{11}H_{21}NO$ was used to detect the elution profile of the analyte.

Powder X-ray diffraction

PXRD measurements were collected on a STOE STADI P instrument at the Integrated Molecular Structure Education and Research Center (IMSERC) at Northwestern University. A CuKα radiation source was used. Samples were prepared by packing MOF powders into metallic flat disc transmission holders and securing the powders using Kapton tape. The ab initio cell indexing has been performed using Dicvol indexing implemented in HighScore Plus 5.2; unit cell parameters were refined by Pawley fitting of the PXRD data.

Elemental analysis

Samples were analyzed using a Thermo iCAP ICP-OES at the Quantitative Bio-element Imaging Center (QBIC) at Northwestern University. For each MOF sample, 2-3 mg (with exact mass measured using an analytical balance) were added to a 2-5 mL Biotage microwave reaction vial,

along with 2 mL nitric acid (trace metal grade, Fisher) and a small stir bar. The microwave vial was then sealed, and the vessel was heated using a Biotage Initiator+ microwave reactor at 150 °C for 15 min until a transparent solution was obtained (Note: additional heating was required to fully digest some samples and obtain a transparent solution). 0.3 mL of the resulting solution was then diluted to a total volume of 10 mL using millipore water.

Scanning electron microscopy

Images of the materials were collected with a Hitachi SU8030 cFEG SEM at the Electron Probe Instrumentation Center (EPIC) facility, which is part of the Northwestern University Atomic and Nanoscale Characterization Experimental Center (NUANCE). Prior to imaging, the samples were deposited onto double-sided carbon tape as dry powders and coated with a 10 nm layer of osmium using an osmium coater (SPI OPC-60A). X-rays microanalysis were performed in a Zeiss Sigma HD microscope coupled to an EDX detector (from Oxford Instruments, x-act PentaFET Precision), working in energy dispersive mode.

Transmission electron microscopy

The morphology and microstructure of the samples were characterized by transmission electron microscopy (TEM) and high-angle annular dark-field (HAADF) scanning transmission electron microscopy (HAADF-STEM) using TEM JEOL F200. Elemental analysis and mapping were performed using a JEOL 100 mm^2 silicon drift energy dispersive X-ray spectrometer (EDX). Carbon supported copper grids, 400 mesh size, were used for preparation of the samples.

Infrared Spectroscopy

FT-IR absorption spectra were carried out using a FT-IR Perkin-Elmer, model 1720X spectrophotometer, at a nominal resolution of 2 cm^{-1}, averaging 100 scans. Measurements were collected using an ATR accessory.

Thermogravimetric analysis

Thermogravimetric analysis (TGA) was performed using a TA Instruments Q500 at a ramp rate of 10 °C/min from 30 to 600 °C under a N_2 flow or air flow of 20 sccm.

X-ray photoelectron spectroscopy

XPS measurements were carried out on a Thermo Scientific ESCALAB 250Xi instrument, which was available through the Keck-II facility, a part of the Northwestern University Atomic and Nanoscale Characterization Experimental Center (NUANCE). Samples were deposited as dry powders onto double-sided copper tape. Monochromatic X-ray source (AlKα), 500 µm spot size and a pass energy of 50 eV were used. Sample charging was prevented with a flood gun. Data analysis was performed with Thermo Avantage software, utilizing the built-in fitting routine. A Smart (constrained Shirley) background was used for all spectra.

Gas sorption isotherms

N_2 isotherms for ZrCo-MOF were collected on a Micromeritics Tristar II 3020 at 77 K. C_2H_2 and C_2H_4 isotherms for ZrCo-MOF were collected on a Micromeritics 3Flex at 298 K. Samples were

first activated under vacuum using a Micromeritics Smart VacPrep 8 instrument at 100 °C for 12-24 h. Prior to activation, samples were thoroughly solvent exchanged by washing with acetone 3× for 1 h and once overnight. Acetylene is supplied dissolved in acetone, so a column of activated carbon and zeolite 13X was used as a solvent trap to prevent solvent contamination in the instrument.[5] Henry's adsorption constants for C_2H_2 and C_2H_4 were calculated from linear fitting of the linear (low pressure) region of the C_2H_2 and C_2H_4 isotherms.

First-principles calculations

All density functional theory (DFT) calculations were carried out using Gaussian 16.[8] Geometry optimizations and vibrational frequency analyses were performed using the B3LYP hybrid functional[9] with ultrafine integration grids, combined with the 6-31+G(d,p) basis set.[10] We chose the B3LYP functional because it produced an optimized structure of the starting material that closely matches that obtained with the state-of-the-art PBE0 functional[11] (Figure S16), while being an order of magnitude faster in computing speed. Harmonic vibrational analyses were employed to confirm the nature of all stationary points on the potential energy surface (minima and transition states) and to compute thermal corrections under standard conditions (298.15 K, 1 atm). Single-point energy calculations were performed for all species to refine their energies, substituting the 6-31+G(d,p) basis set with the more accurate 6-311++G(d,p) basis set.[12] The reported Gibbs free energy of each species includes the sum of its electronic energy at the 6-311++G(d,p) level and its thermodynamic correction at the 6-31+G(d,p) level. This combination of functional and basis sets, noted for its effective performance-to-cost ratio, has reliably described the geometric and electronic structures in previous computational studies of relevant systems.[13] The conductor-like polarizable continuum model (CPCM)[14] with Bondi radii[15] was used to

model solvation effects implicitly within the acetonitrile solution. In this model, the overlap index for two interlocking spheres on the solvent-excluding surface was set to 0.8, and the minimum radius for these solvent-excluding surface spheres was set to be 0.5 Å.

Efforts to identify a transition state for the reaction of *Co-H + C_2H_2 → *Co-$CHCH_2$ proved challenging. Scanning of the potential energy surface (PES) along the hydrogen atom transfer (HAT) pathway has been conducted. This includes a hydrogen transfer from *Co to C_2H_2, where the energy change was examined as the distance between the hydrogen and one of the two carbon sites in C_2H_2 decreased from 2.5 to 1.0 Å (Figure S17a). Alternatively, the scan was also conducted for a hydrogen in the CH_2 group of CH_2CH transferring back to *Co, with the energy change observed as the distance between this hydrogen and the Co site decreased from 3.0 to 1.8 Å (Figure S17b). However, both PES scans revealed no discernible energy barriers. The absence of an energy barrier on the PES, coupled with the highly exothermic nature of this transition (a substantial decrease of 17.9 kcal/mol), suggests a barrierless HAT process for this step. A barrierless HAT has been observed in another similar cobalt complex, Co^{II}(*t*-Bu,*t*-Bu-cyclohexylsalen).[16]

Supplementary Figures

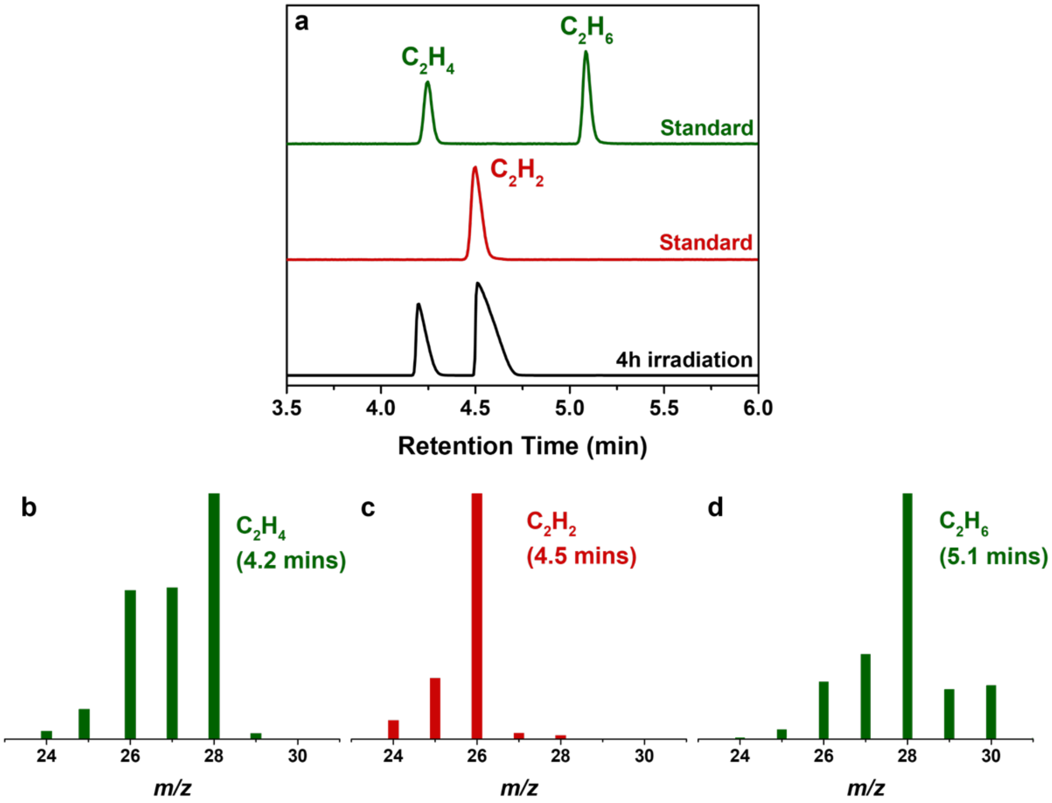

Figure S1. Typical GC-MS chromatograms and mass spectra observed for the photoreduction of acetylene. **(a)** Gas chromatograms of the C_2H_4/C_2H_6 standard (green), C_2H_2 standard (red), and the C_2H_2 (≥99.5 vol.%)-purged acetonitrile solution containing 2.5 mM $[Ru(bpy)_3]^{2+}$, 1.0 mM $[Co(dmgH)_2pyCl]$, 1.0 M TFE and 0.1 M BIH after irradiation with 450 nm light (140 $mW{\cdot}cm^{-2}$) for 4 h (black). **(b)** Mass spectrum of C_2H_4. **(c)** Mass spectrum of C_2H_2. **(d)** Mass spectrum of C_2H_6.

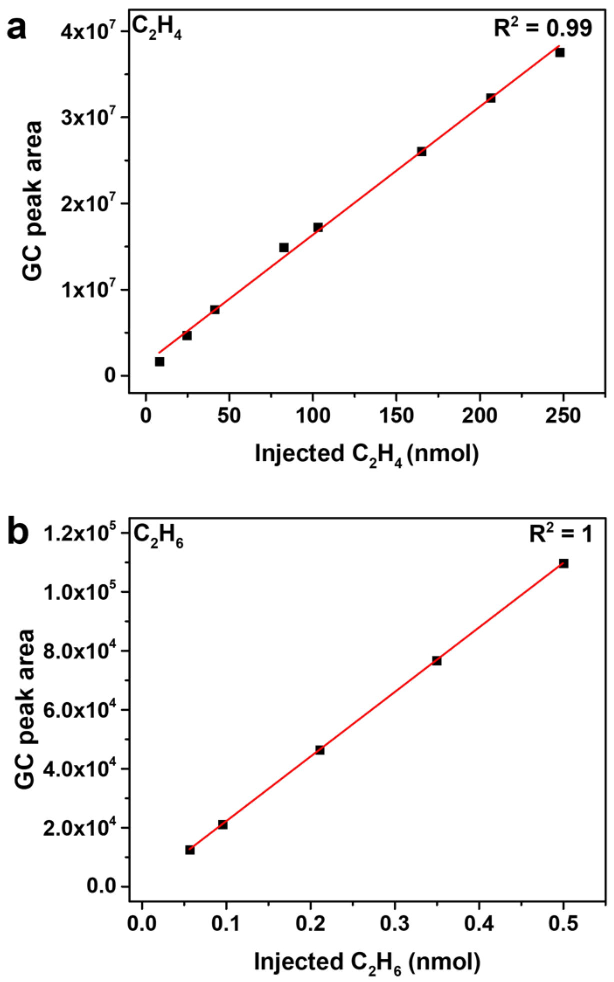

Figure S2. Calibration curves for quantification of gaseous products of the photoreduction of acetylene using GC-MS. **(a)** Calibration curve for C_2H_4 with the corresponding coefficient of linear correlation (R^2). **(b)** Calibration curve for C_2H_6 with the corresponding coefficient of linear correlation (R^2).

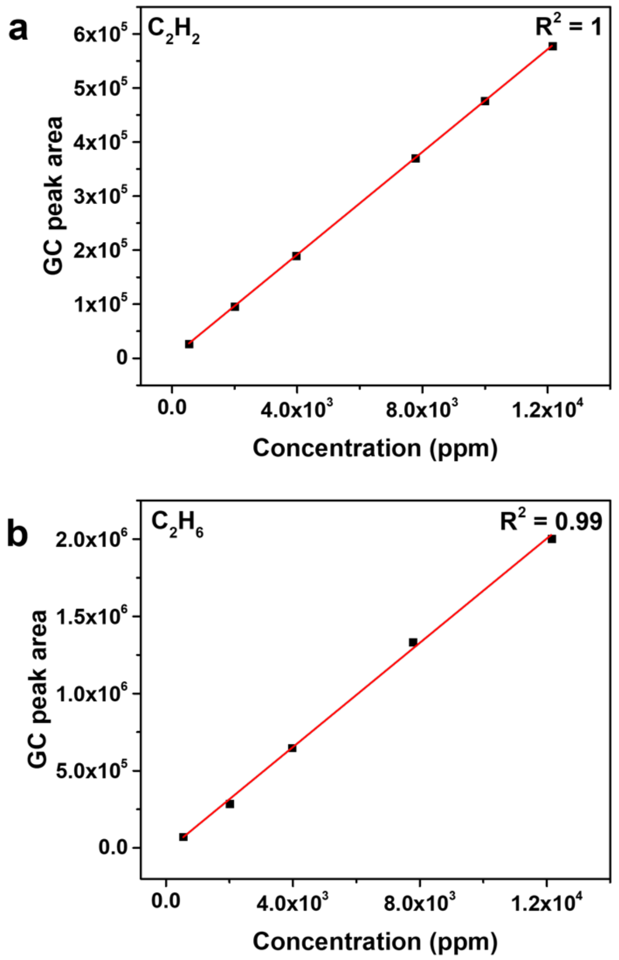

Figure S3. Calibration curves for quantification of gaseous products of the photoreduction of the acetylene/ethylene industrial mixture using GC-FID. **(a)** Calibration curve for C_2H_2 with the corresponding coefficient of linear correlation (R^2). **(b)** Calibration curve for C_2H_6 with the corresponding coefficient of linear correlation (R^2).

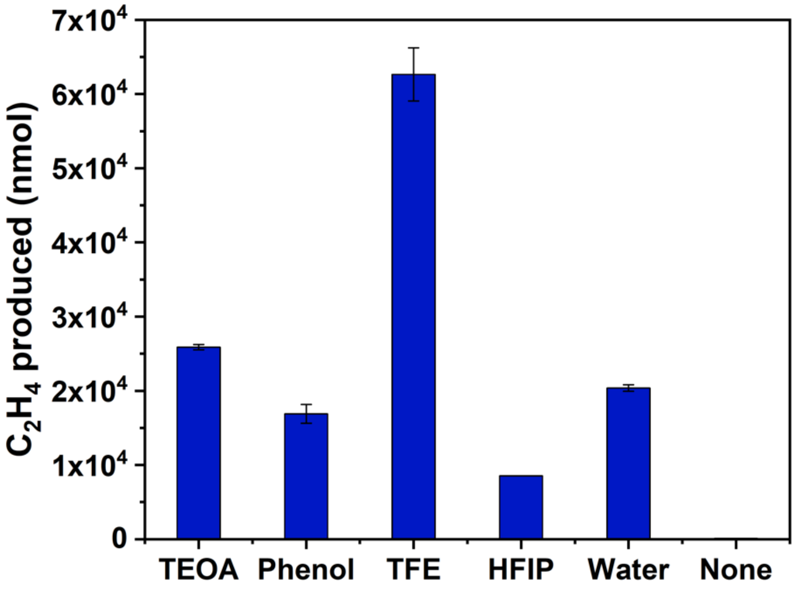

Figure S4. Photocatalytic performance through variation of sacrificial donor/proton donor. Optimization of amount of C_2H_4 (nmol C_2H_4) produced by the $[Co(dmgH)_2pyCl]/[Ru(bpy)_3]^{2+}$ system under C_2H_2 (≥99.5 vol.%) after irradiation with 450 nm light (140 mW·cm^{-2}) for 4 h in acetonitrile through variation of 1.0 M TEOA or Phenol or TFE or HFIP, or in acetonitrile/water (6:1 *v*/*v*), and in the presence of 2.5 mM $[Ru(bpy)_3]^{2+}$, 1.0 mM $[Co(dmgH)_2pyCl]$ and 0.1 M BIH. Error bars indicate standard error of the mean, calculated from two to three runs.

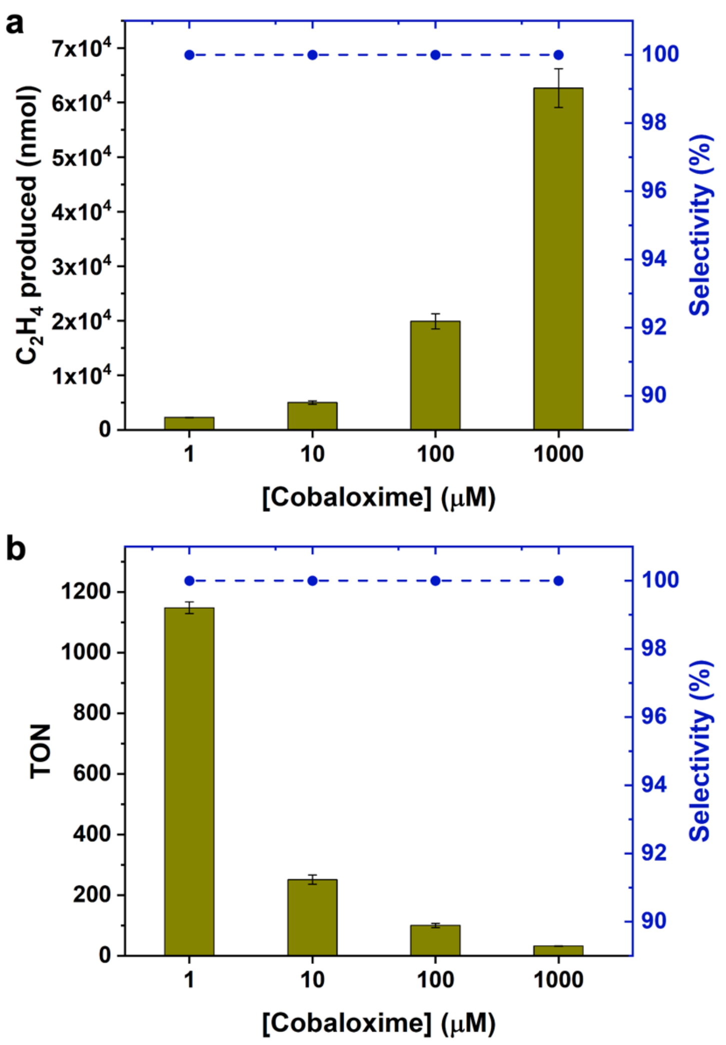

Figure S5. Photocatalytic performance through variation of [Cobaloxime]. Optimization of **(a)** amount of C_2H_4 (nmol C_2H_4) produced, and **(b)** TON (C_2H_4) and selectivity for C_2H_4 by the [Co(dmgH)$_2$pyCl]/[Ru(bpy)$_3$]$^{2+}$ system in acetonitrile under C_2H_2 (≥99.5 vol.%) after irradiation with 450 nm light (140 mW·cm^{-2}) for 4 h through variation of [[Co(dmgH)$_2$pyCl]] in the presence of 2.5 mM [Ru(bpy)$_3$]$^{2+}$, 1.0 M TFE and 0.1 M BIH. Error bars indicate standard error of the mean, calculated from two to three runs.

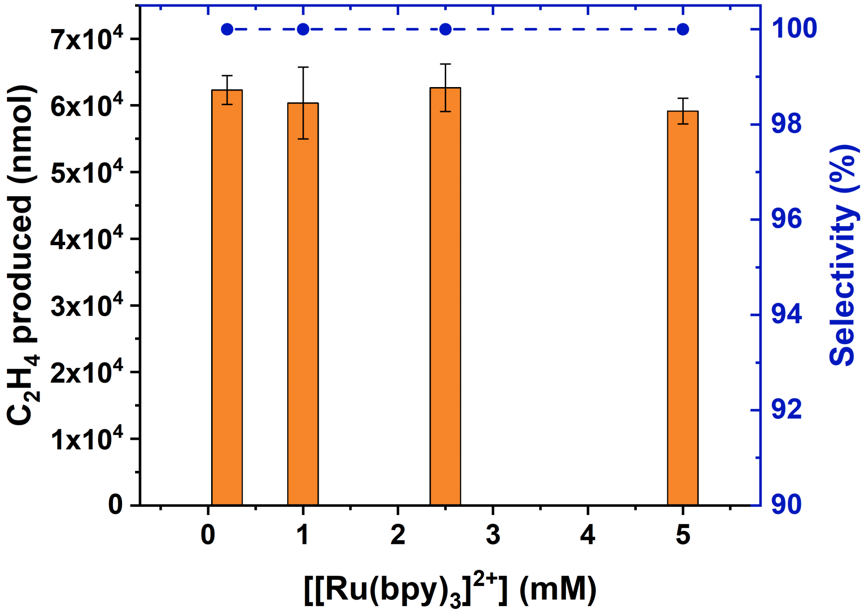

Figure S6. Photocatalytic performance through variation of [[Ru(bpy)$_3$]$^{2+}$]. Optimization of amount of C_2H_4 (nmol C_2H_4) produced and selectivity for C_2H_4 by the [Co(dmgH)$_2$pyCl]/[Ru(bpy)$_3$]$^{2+}$ system in acetonitrile under C_2H_2 (≥99.5 vol.%) after irradiation with 450 nm light (140 mW·cm^{-2}) for 4 h through variation of [[Ru(bpy)$_3$]$^{2+}$] in the presence of 1.0 mM [Co(dmgH)$_2$pyCl], 1.0 M TFE and 0.1 M BIH. Error bars indicate standard error of the mean, calculated from two to three runs.

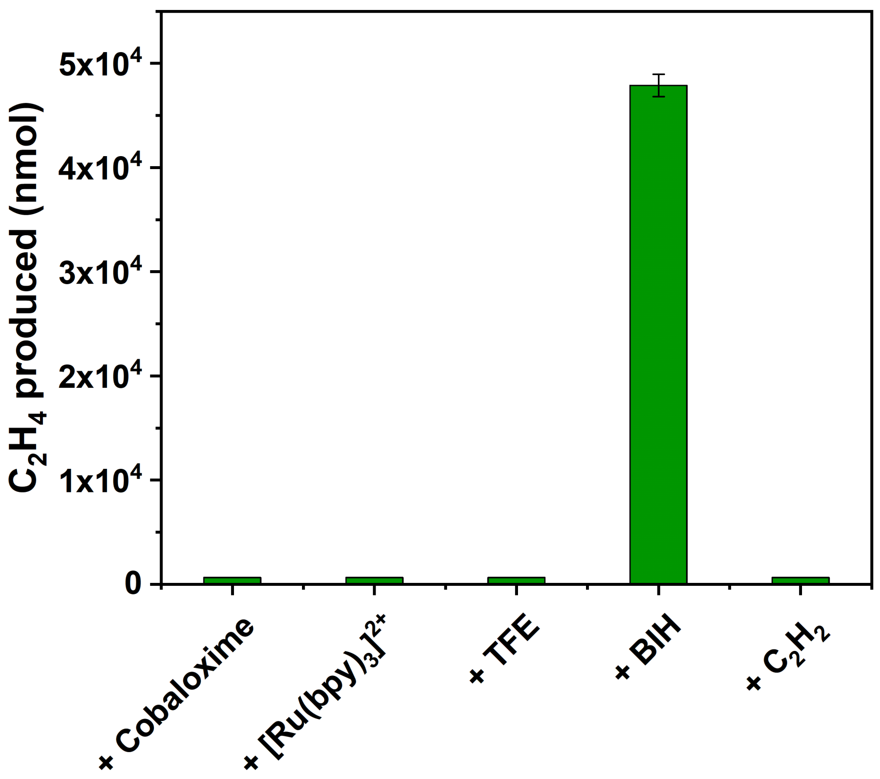

Figure S7. Re-addition experiments showing recovery of C_2H_4 production. Amount of C_2H_4 (nmol C_2H_4) produced by the [Co(dmgH)$_2$pyCl]/[Ru(bpy)$_3$]$^{2+}$ system in acetonitrile under C_2H_2 (≥99.5 vol.%) first irradiated with 450 nm light (140 mW·cm^{-2}) for 20 h and then further irradiated for an additional 4 h upon re-addition of 1.0 mM [Co(dmgH)$_2$pyCl] or 2.5 mM [Ru(bpy)$_3$]$^{2+}$ or 1.0 M TFE or 0.1 M BIH or C_2H_2. Error bars indicate standard error of the mean, calculated from two to three runs.

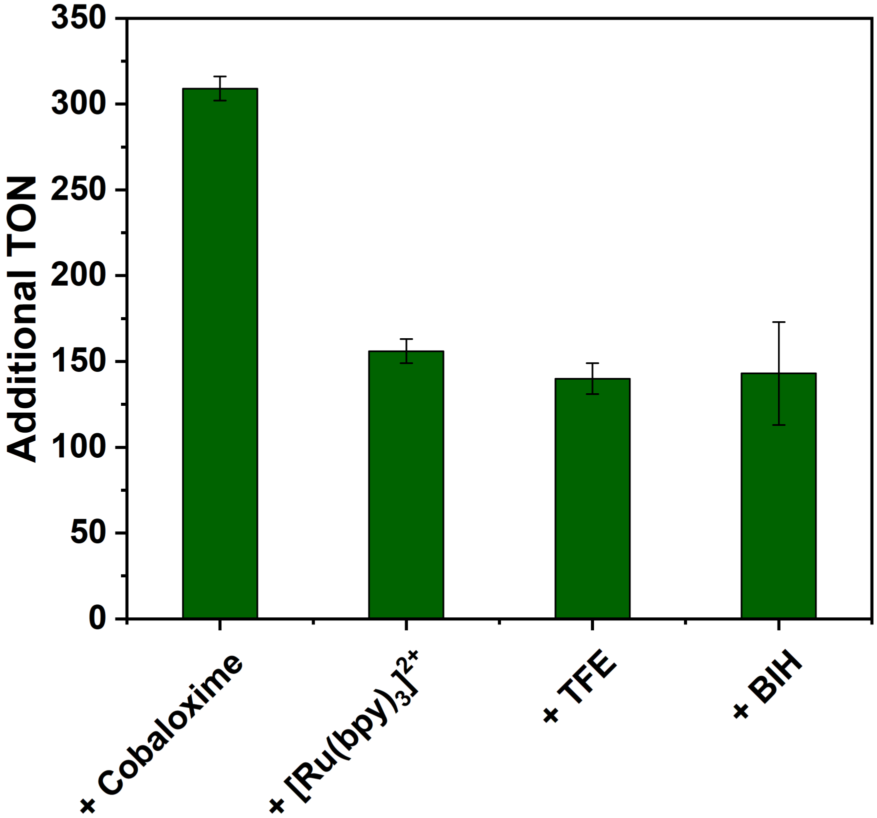

Figure S8. Re-addition experiments showing recovery of C_2H_4 production. TON (C_2H_4) produced by the [Co(dmgH)$_2$pyCl]/[Ru(bpy)$_3$]$^{2+}$ system in acetonitrile under C_2H_2 (≥99.5 vol.%) first irradiated with 450 nm light (140 mW·cm^{-2}) for 20 h and then further irradiated for an additional 8 h upon re-addition of 1.0 μM [Co(dmgH)$_2$pyCl] or 2.5 mM [Ru(bpy)$_3$]$^{2+}$ or 1.0 M TFE or 0.1 M BIH. Error bars indicate standard error of the mean, calculated from two to three runs.

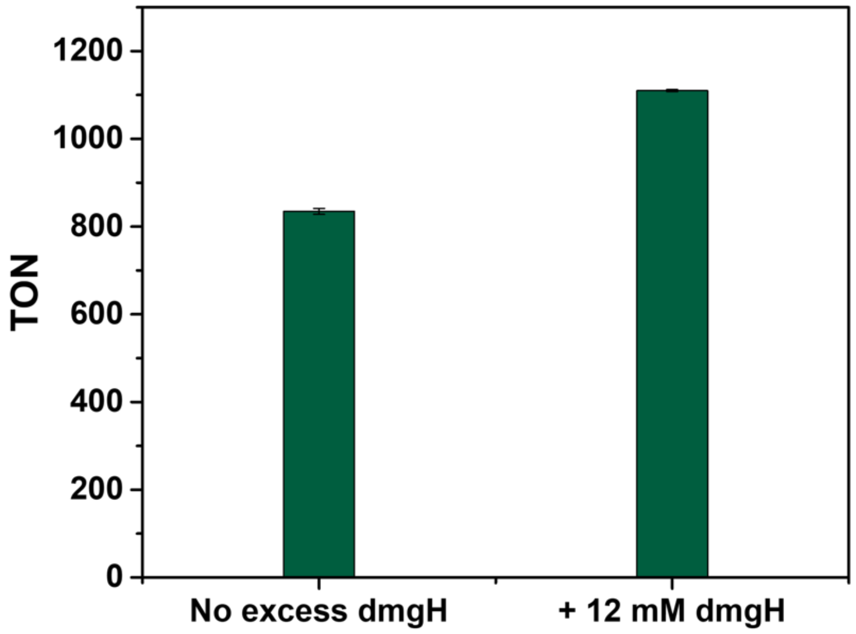

Figure S9. Photocatalytic performance in presence of excess dimethylglyoxime ligand. TON (C_2H_4) by the [Co(dmgH)$_2$pyCl]/[Ru(bpy)$_3$]$^{2+}$ system in acetonitrile under C_2H_2 (≥99.5 vol.%) after irradiation with 450 nm light (140 mW·cm^{-2}) for 4 h containing 1.0 μM [Co(dmgH)$_2$pyCl], 10 μM [Ru(bpy)$_3$]$^{2+}$, 1.0 M TFE and 0.1 M BIH, with and without 12 mM dmgH ligand. Error bars indicate standard error of the mean, calculated from two to three runs.

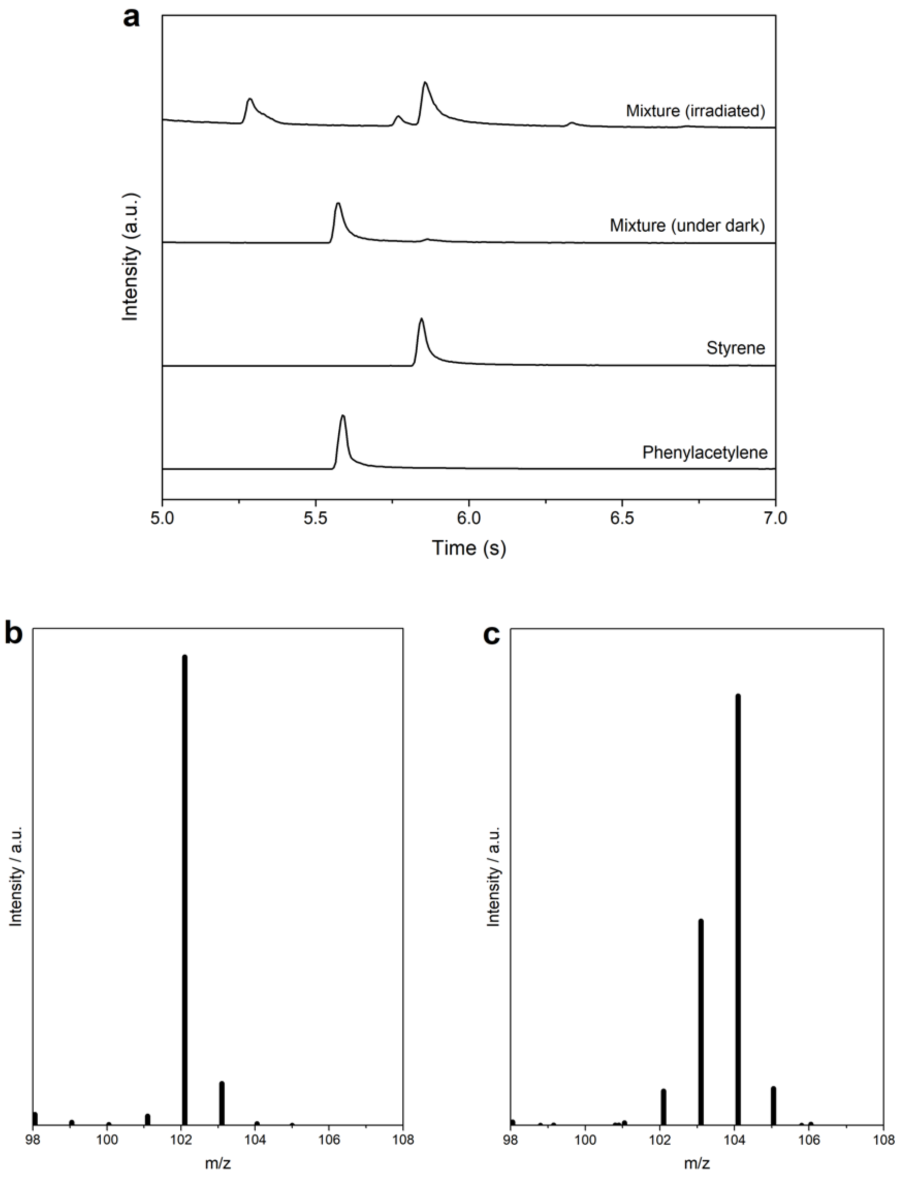

Figure S10. Typical GC-MS chromatograms and mass spectra observed for the photoreduction reaction of phenylacetylene. **(a)** Gas chromatograms of an acetonitrile solution containing 2.5 mM $[Ru(bpy)_3]^{2+}$, 10 mol% $[Co(dmgH)_2pyCl]$, 1.0 M TFE, 0.1 M BIH and 10 mM phenylacetylene after kept in the dark or after irradiation with 450 nm light (140 $mW\cdot cm^{-2}$) for 18 h, styrene standard and phenylacetylene standard. **(b)** Mass spectrum of phenylacetylene. **(c)** Mass spectrum of styrene.

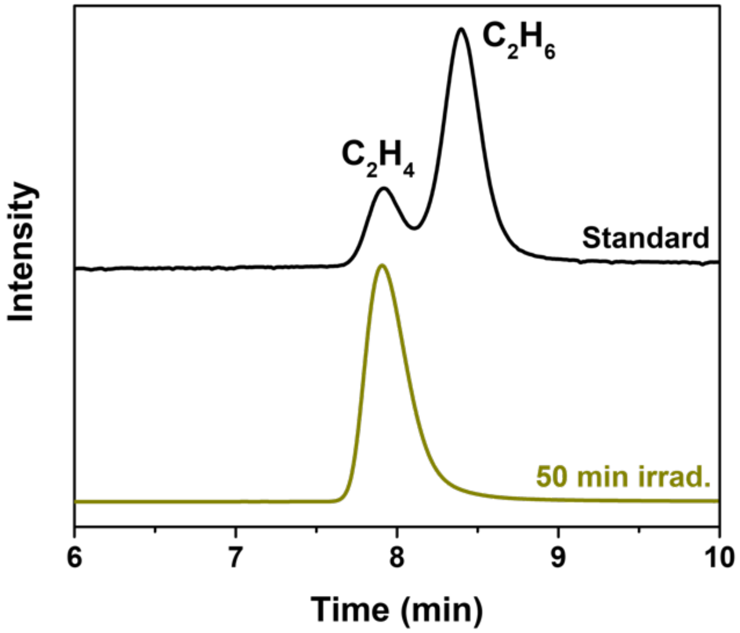

Figure S11. Typical GC-FID chromatograms for the photoreduction of the acetylene/ethylene mixture. Gas chromatograms of the C_2H_4/C_2H_6 standard (black), and the [Co(dmgH)$_2$pyCl]/[Ru(bpy)$_3$]$^{2+}$ system in acetonitrile containing 2.5 mM [Ru(bpy)$_3$]$^{2+}$, 1.0 mM [Co(dmgH)$_2$pyCl], 1.0 M TFE and 0.1 M BIH under C_2H_2/C_2H_4 (1 vol.% C_2H_2, 30 vol.% C_2H_4, He balance) mixture after irradiation with 450 nm light (140 mW·cm^{-2}) for 50 min (green).

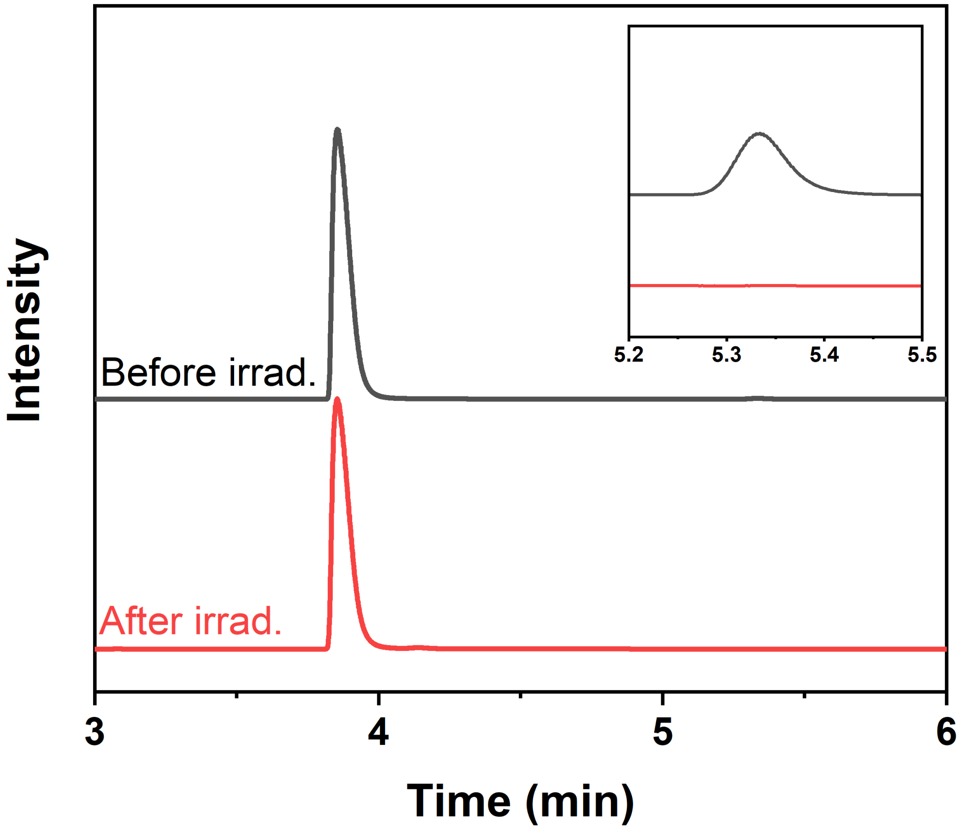

Figure S12. Typical GC-FID chromatograms for the photoreduction of the acetylene/ethylene mixture. Gas chromatograms of the [Co(dmgH)$_2$pyCl]/[Ru(bpy)$_3$]$^{2+}$ system in acetonitrile containing 2.5 mM [Ru(bpy)$_3$]$^{2+}$, 1.0 mM [Co(dmgH)$_2$pyCl], 1.0 M TFE and 0.1 M BIH under C_2H_2/C_2H_4 (1 vol.% C_2H_2, 99 vol.% C_2H_4) mixture before (grey) and after irradiation with 450 nm light (140 mW·cm^{-2}) for 34 min (red).

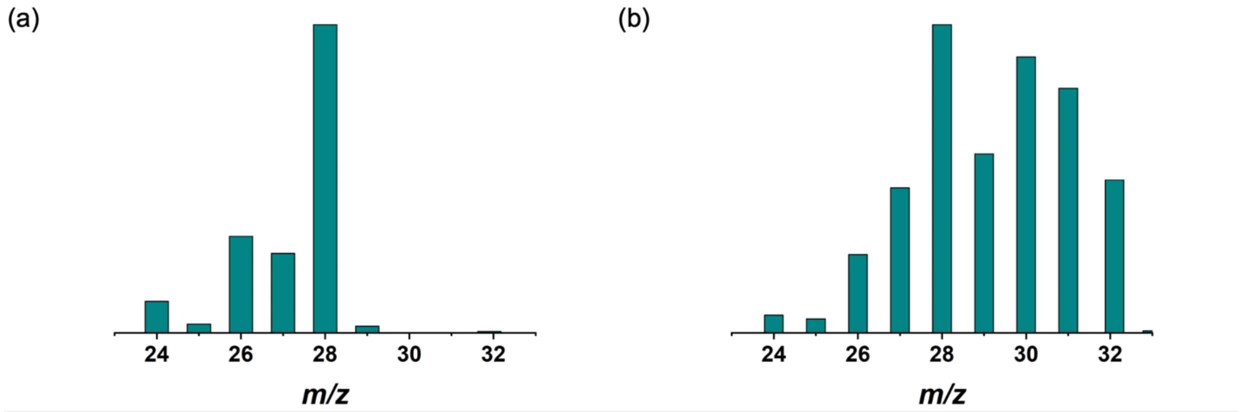

Figure S13. GC-MS spectra of **(a)** acetylene feedstock and **(b)** ethylene product for photocatalytic systems using 2.5 mM $[Ru(bpy)_3]^{2+}$, 1.0 mM $[Co(dmgH)_2pyCl]$, and 0.1 M BIH in trifluoroethanol-d_3/acetonitrile (3:1 *v*/*v*, 2 mL) irradiated (450 nm, 140 $mW \cdot cm^{-2}$) for 4 h under C_2H_2 (5 vol.%). The system was left to pre-equilibrate for 4 h before irradiation. Starting with **(a)** C_2D_2 it is possible to observe a shift $m/z + 4$ that corresponds to incorporation of two deuteriums to produce **(b)** C_2D_4.

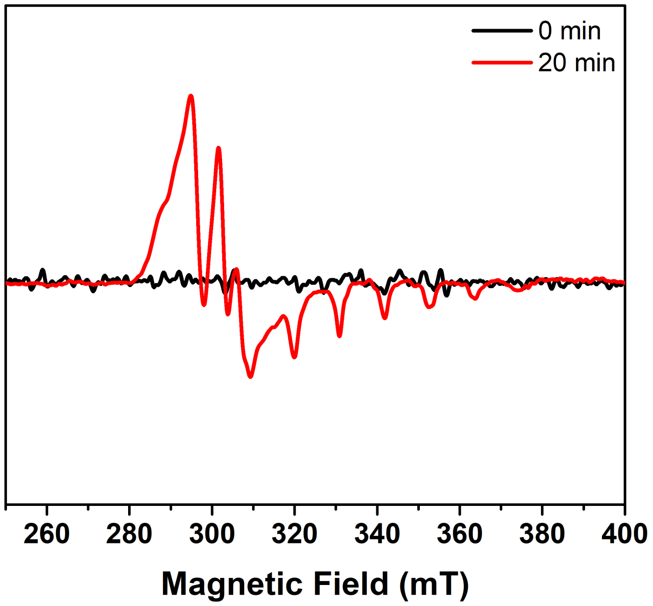

Figure S14. Electron paramagnetic resonance spectra of [Co(dmgH)$_2$pyCl] in acetonitrile (77 K) under inert atmosphere, recorded immediately after mixing with BIH (0 min) and after waiting (20 min) at room temperature. We noticed, also when performing UV-vis experiments, that Co^{III} is not immediately fully reduced to Co^{II} but it is necessary to wait to appreciate its formation.

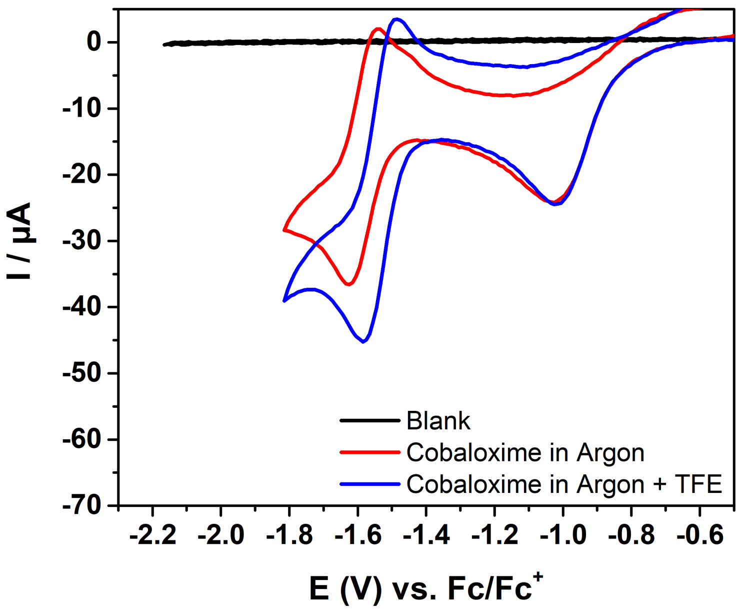

Figure S15. Cyclic voltammograms of $[Co(dmgH)_2pyCl]$ with and without TFE in acetonitrile supported with TBA•PF_6 and saturated with Ar.

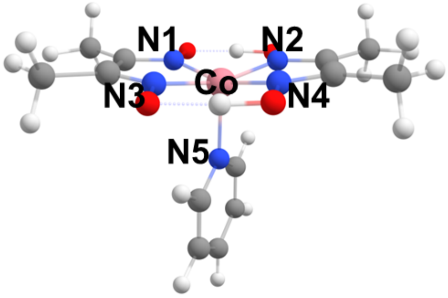

Bond length (Å)	Co-N1	Co-N2	Co-N3	Co-N4	Co-N5
PBE0	1.86	1.84	1.86	1.84	2.01
B3LYP	1.89	1.87	1.89	1.87	2.06

Figure S16. Comparison of the key bond lengths optimized using PBE0 and B3LYP functionals for the starting material.

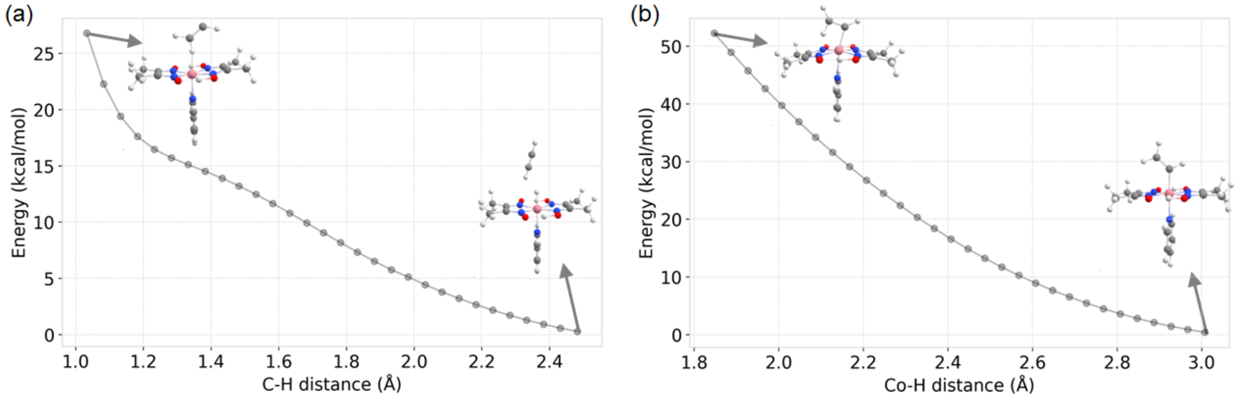

Figure S17. Potential energy surface scan along the HAT pathway, initiated from **(a)** C_2H_2 adsorption on the *Co-H site to examine the energy change as the hydrogen on *Co transfers to one of the two carbon sites in C_2H_2 and **(b)** *Co-CHCH$_2$ to examine the energy change as the hydrogen in the CH_2 group of CH_2CH transfers back to *Co.

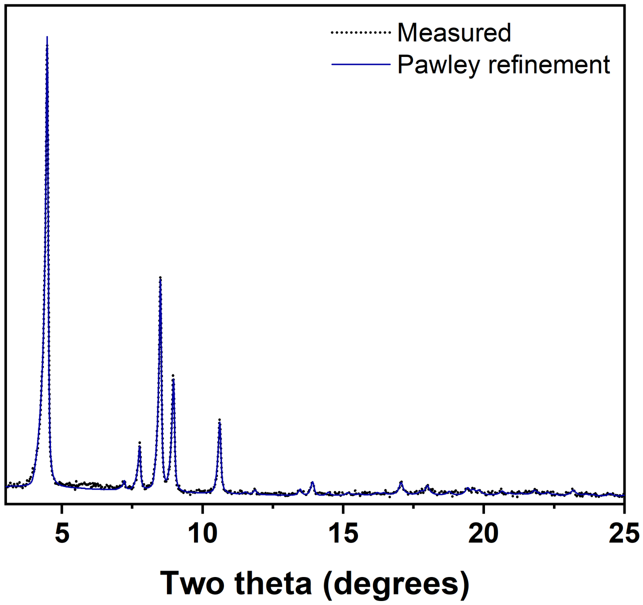

Figure S18. PXRD pattern of pristine ZrCo-MOF (black). Pawley refinement of PXRD measurement (blue): space group *P*222; R_{wp} = 6.913%, R_p = 5.474%, GOF = 1.170; *a* = 19.70(1) Å, *b* = 12.226(2) Å, *c* = 11.378(3) Å.

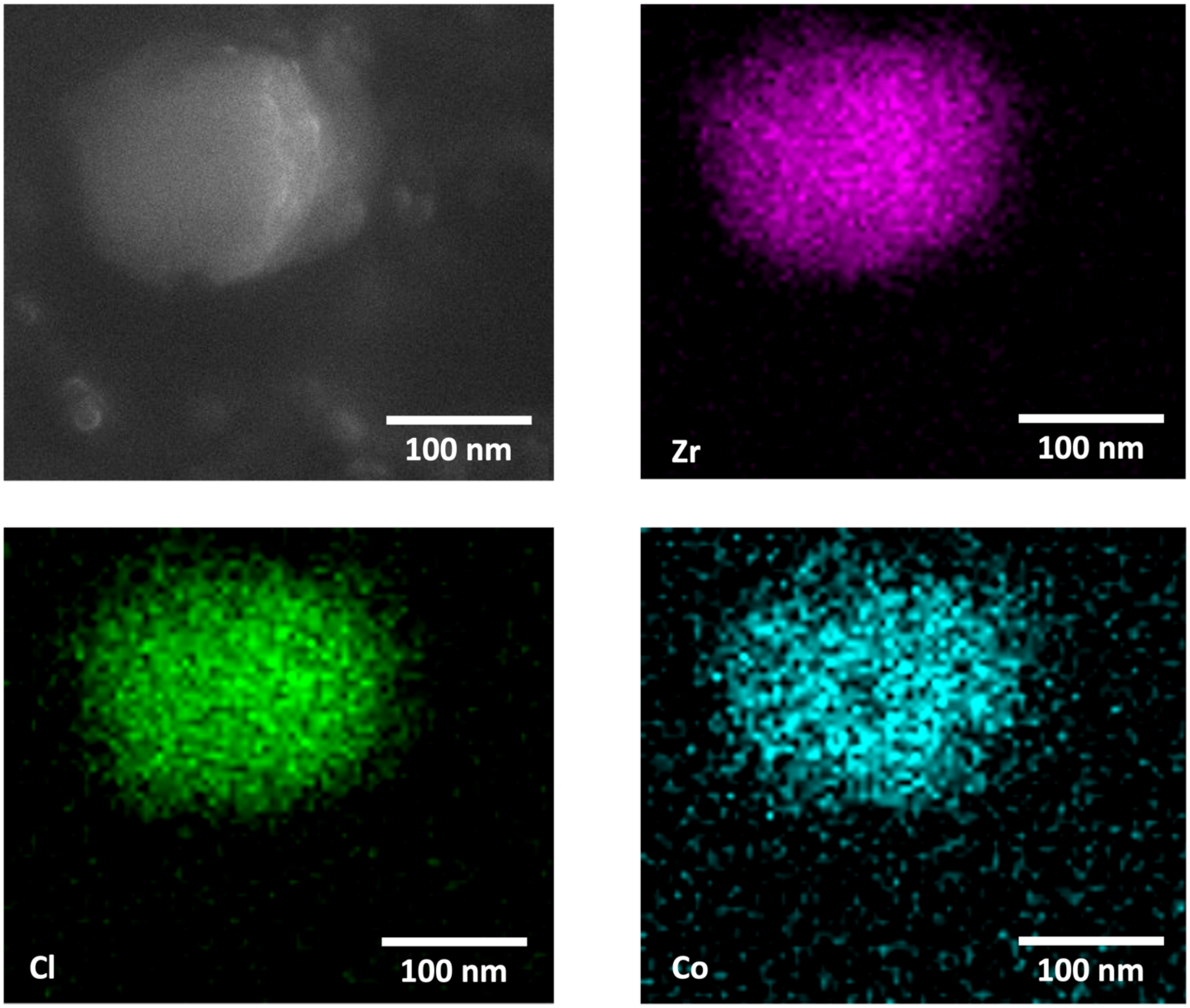

Figure S19. SEM images of ZrCo-MOF with corresponding energy dispersive X-ray spectroscopy elemental maps of Zr Lα, Co Kα, and Cl Kα.

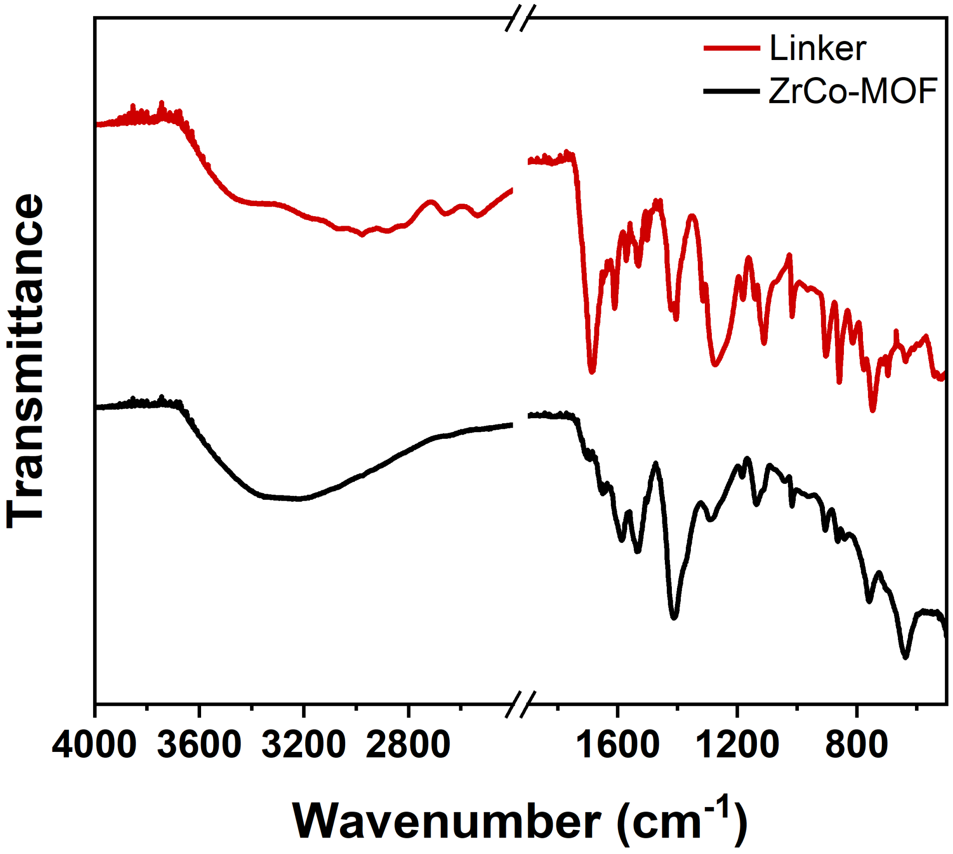

Figure S20. ATR-IR spectra of [Co(dcpgH)(dcpgH$_2$)Cl$_2$] (red) and ZrCo-MOF (black). ZrCo-MOF spectrum shows Zr-μ_3-O stretch at 640 cm^{-1}, COO symmetric stretch at 1411 cm^{-1}, C=N oxime stretch at 1586 cm^{-1}, COO asymmetric stretch at 1655 cm^{-1}.

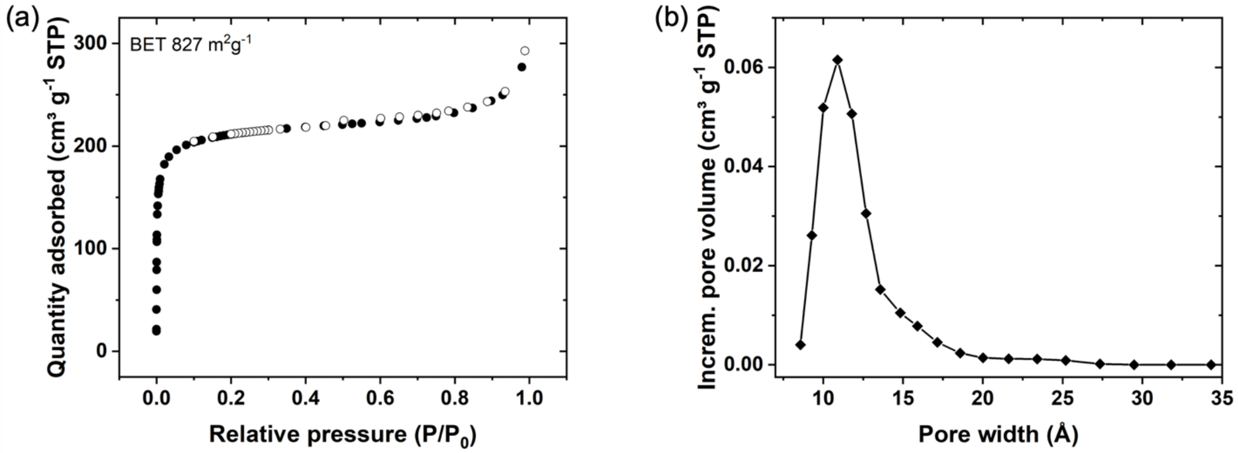

Figure S21. **(a)** N_2-sorption isotherm of ZrCo-MOF, and **(b)** pore size distribution.

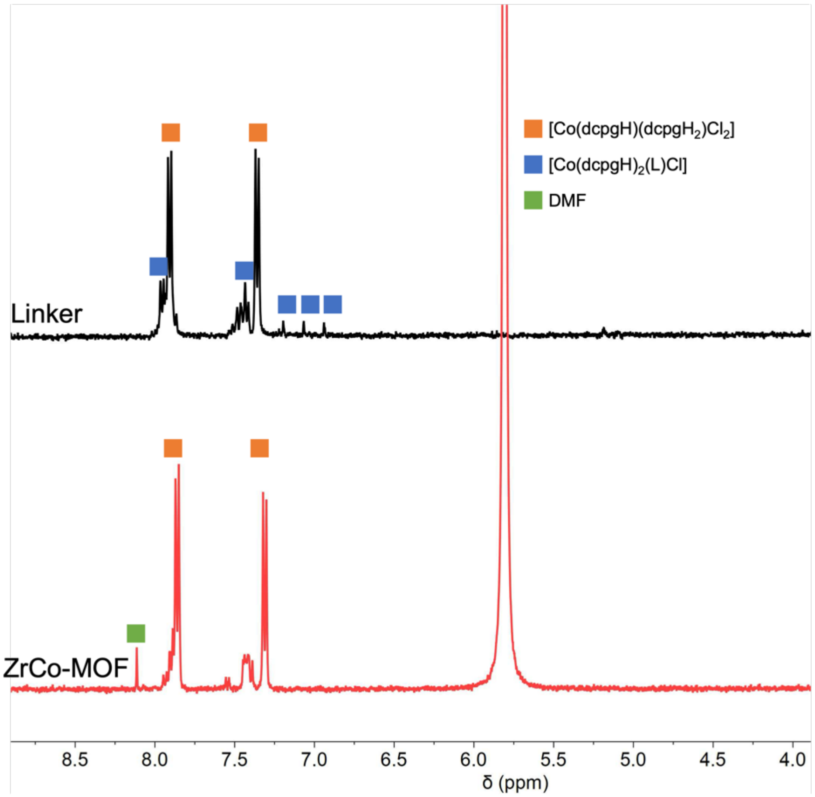

Figure S22. ^{1}H-NMR spectra of linker [Co(dcpgH)(dcpgH$_2$)Cl$_2$] (black trace) and digested ZrCo-MOF (red trace) in DMSO-d_6. 2.5 mg ZrCo-MOF were digested by heating at 50 °C for 2 h in 0.6 mL DCl/DMSO-d_6 (1:10 *v/v*).

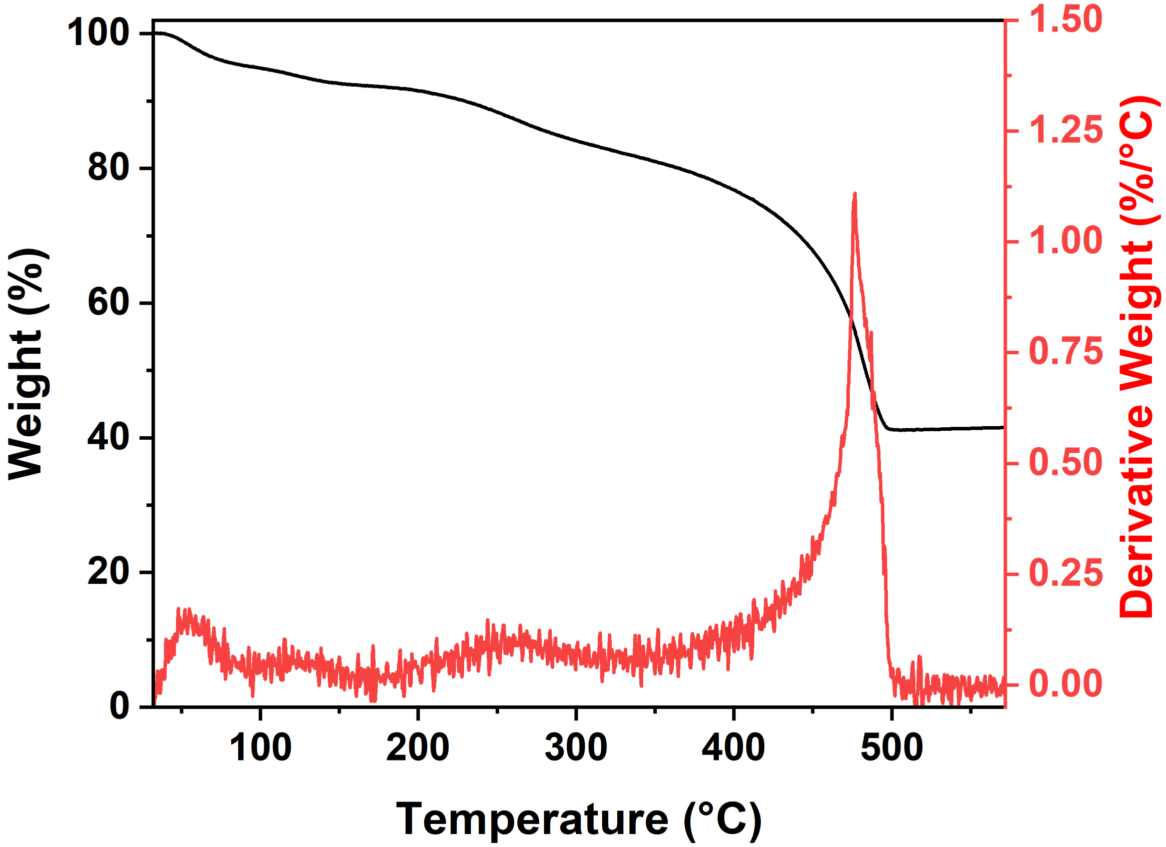

Figure S23. TGA of ZrCo-MOF.

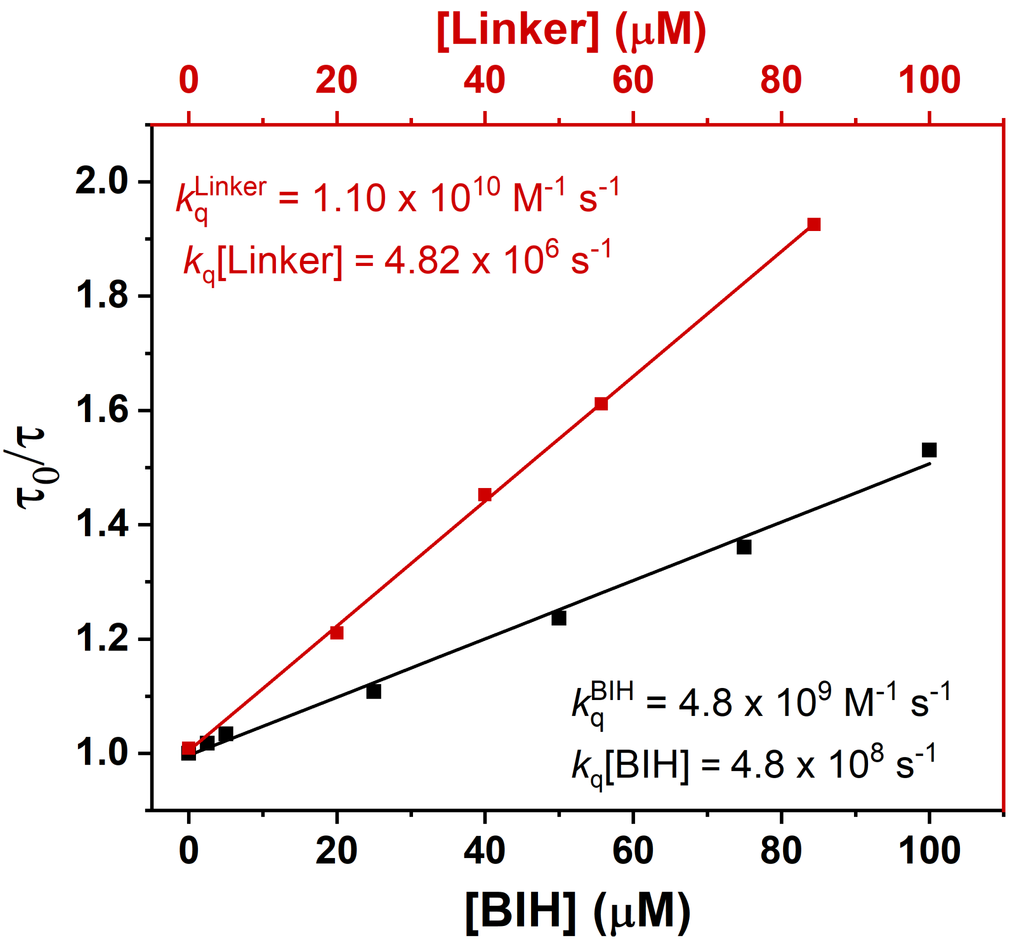

Figure S24. Stern-Volmer plots for the quenching of $[Ru(bpy)_3]^{2+}$ emission lifetime by $[Co(dcpgH)(dcpgH_2)Cl_2]$ (red) or BIH (black).

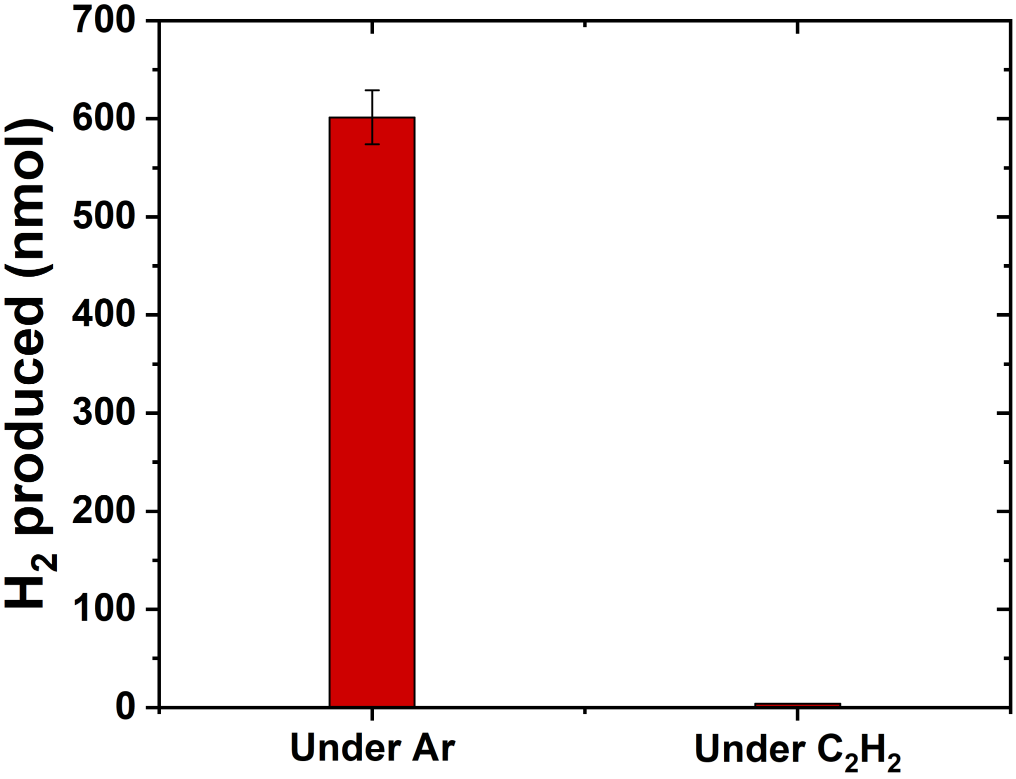

Figure S25. H_2 evolution by the ZrCo-MOF-based system for the reaction mixture containing 2.5 mM $[Ru(bpy)_3]^{2+}$, 1.0 mg ZrCo-MOF, 1.0 M TFE and 0.1 M BIH in acetonitrile irradiated (450 nm, 140 mW·cm^{-2}) for 4 h under Ar vs C_2H_2 (≥99.5 vol.%). Error bars indicate standard error of the mean, calculated from two to three runs.

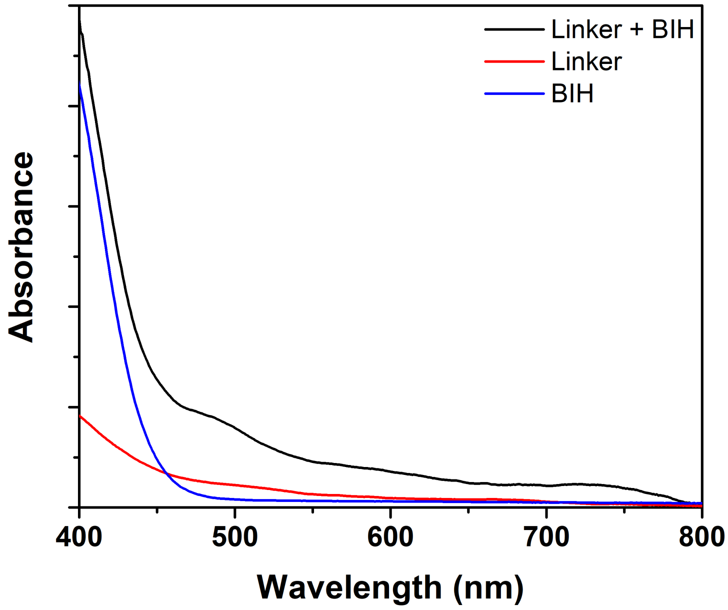

Figure S26. UV-vis spectra of 100 μM [Co(dcpgH)(dcpgH_2)Cl_2] in acetonitrile under inert atmosphere, without (red) and with (black) 0.1 M BIH.

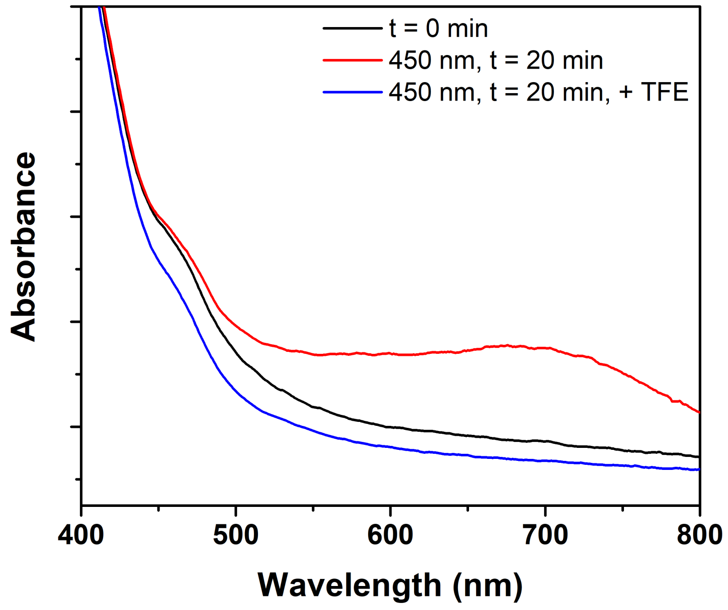

Figure S27. UV-vis spectra of the system containing 100 μM [Co(dcpgH)(dcpgH_2)Cl_2] in acetonitrile under inert atmosphere, 50 μM $[Ru(bpy)_3]^{2+}$, 0.1 M BIH and 1.0 M TFE under various conditions.

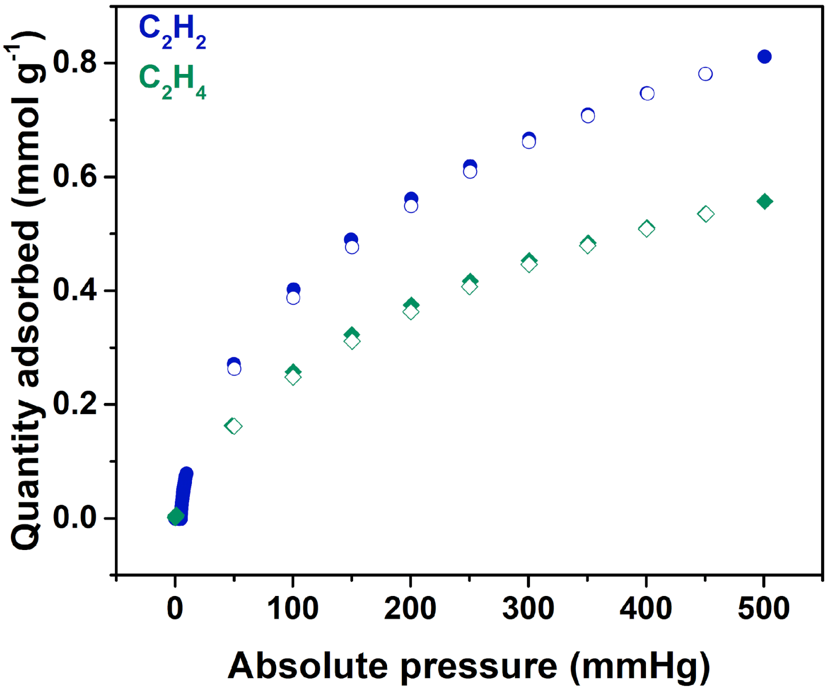

Figure S28. Quantity adsorbed (mmolg^{-1}) vs. absolute pressure (mmHg) of C_2H_2 (blue) and C_2H_4 (green) for ZrCo-MOF collected at 25°C. Filled symbols denote adsorption isotherms, and open symbols desorption isotherms.

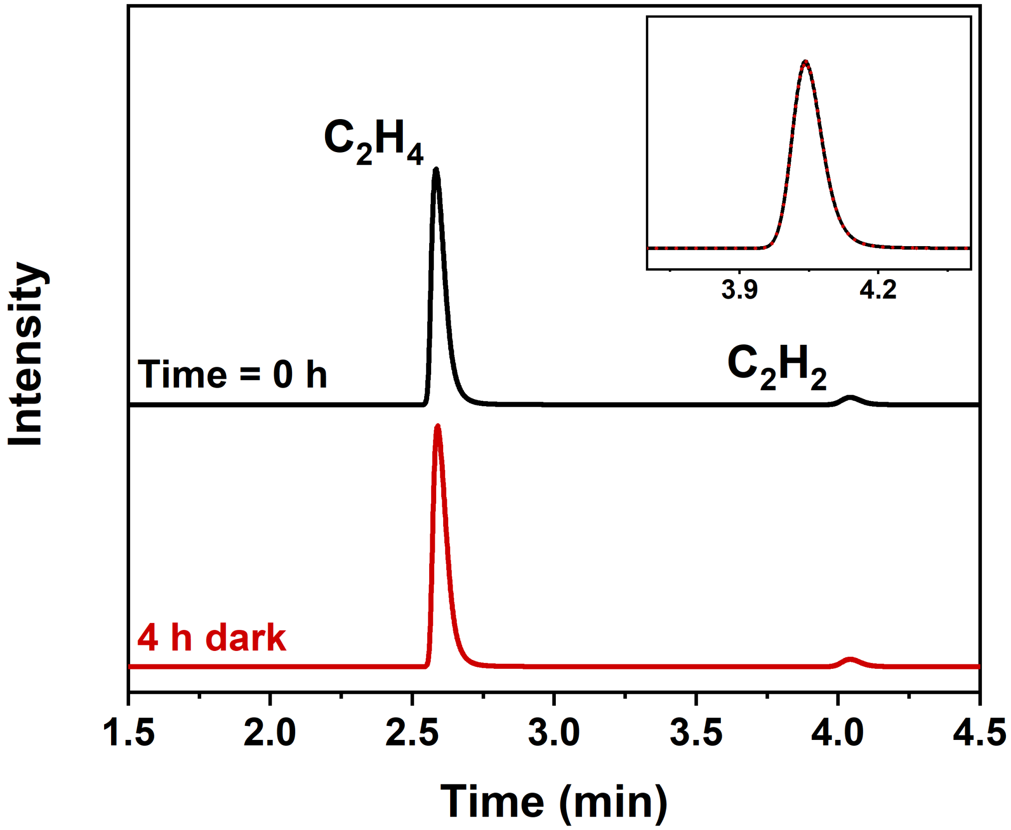

Figure S29. Typical GC-FID chromatograms of the control reactions for the photoreduction of the acetylene/ethylene mixture. Gas chromatograms of the ZrCo-MOF/$[Ru(bpy)_3]^{2+}$ system in acetonitrile containing 2.5 mM $[Ru(bpy)_3]^{2+}$, 2.28 mg ZrCo-MOF, 1.0 M TFE and 0.1 M BIH under C_2H_2/C_2H_4 (1 vol.% C_2H_2, 30 vol.% C_2H_4, He balance) mixture before irradiation (time = 0, black) and after being kept in the dark for 4 h (red).

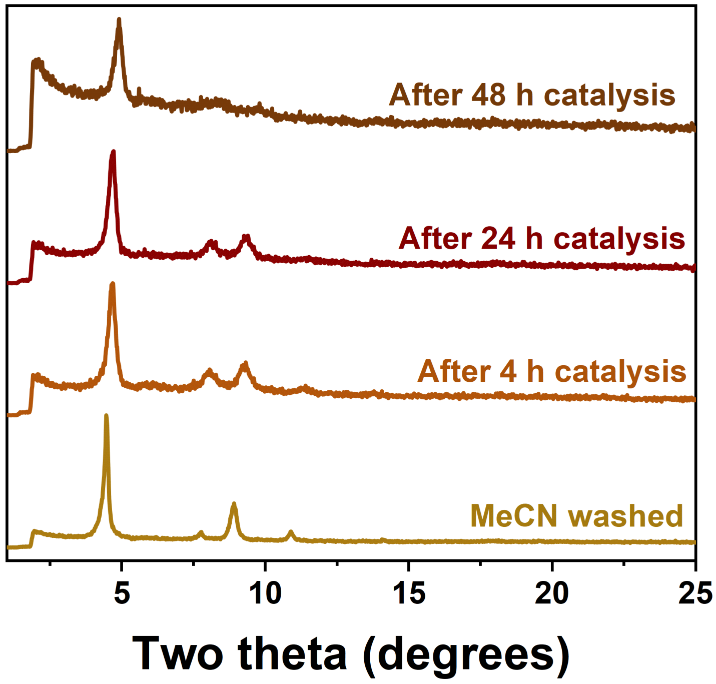

Figure S30. PXRD of ZrCo-MOF. PXRD patterns of ZrCo-MOF before (yellow) and after various reaction times. The samples contained 2.5 mM $[Ru(bpy)_3]^{2+}$, 2.28 mg ZrCo-MOF, 1.0 M TFE and 0.1 M BIH in acetonitrile and were irradiated for 4 h (orange) or 24 h (red) under C_2H_2 (≥99.5 vol.%), and for 48 h (brown) under a C_2H_2/C_2H_4 mixture (1 vol.% C_2H_2, 30 vol.% C_2H_4, He balance).

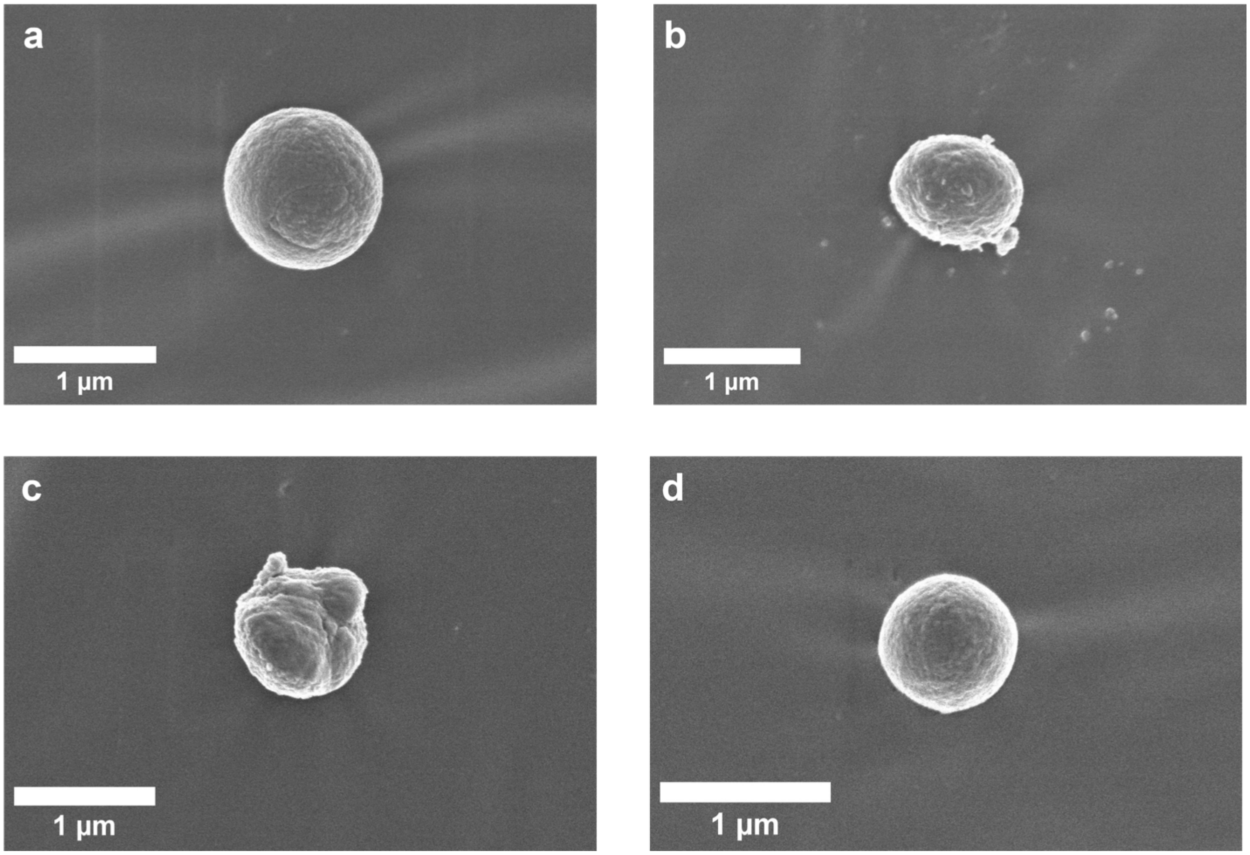

Figure S31. SEM of ZrCo-MOF. SEM images of ZrCo-MOF **(a)** before and after **(b)** 4 h **(c)** 24 h and **(d)** 48 h of photocatalysis. The samples contained 2.5 mM $[Ru(bpy)_3]^{2+}$, 2.28 mg ZrCo-MOF, 1.0 M TFE and 0.1 M BIH in acetonitrile under C_2H_2 (≥99.5 vol.%), except for (d), which corresponds to powder recovered from the industrial mixture recycling experiment, and was thus under a C_2H_2/C_2H_4 mixture (1 vol.% C_2H_2, 30 vol.% C_2H_4, He balance) during illumination.

Supplementary Tables

Table S1. Elemental analysis *via* ICP-OES for pre- and post-catalysis ZrCo-MOF samples.

Sample	Measured [Zr] (mg L^{-1})	Measured [Co] (mg L^{-1})	Moles Zr	Moles Co	Co/Zr_6 Node
Pre-catalysis (0 h)	6.35	1.64	7.07×10^{-7}	2.82×10^{-7}	2.4
Post-catalysis (4 h)	5.33	1.32	5.81×10^{-7}	2.22×10^{-7}	2.3
Post-catalysis (24 h)	5.52	1.39	6.15×10^{-7}	2.40×10^{-7}	2.3
Post-catalysis (48 h)	3.47	0.74	3.86×10^{-7}	1.27×10^{-7}	2.0

Note: The absolute concentrations of Zr (Measured [Zr]) and Co (Measured [Co]) reflect a different amount of MOF digested in each sample. The Co/Zr ratio (Co atoms per Zr_6 node, Co/Zr_6 Node) does not depend on the amount of MOF digested to prepare the samples, and is thus a pure reflection of the number of Co atoms per node. Using this value, it is observed only a small loss in post-catalysis samples compared with the pre-catalysis sample.

Table S2. Photocatalytic and control reactions for C_2H_2 to C_2H_4 photoreduction.

Entry	Catalyst (mM or mg)	C_2H_4 (mmol)	Sel. C_2H_4 vs. C_2H_6 (%)
**1**	[Co(dmgH)$_2$pyCl] Sigma-Aldrich (1 mM)	63 ± 3	>99.9
**2**	[Co(dmgH)$_2$pyCl] Sigma-Aldrich (0.44 mM)	46 ± 3	>99.9
**3**	[Co(dmgH)$_2$pyCl] our synthesis (1 mM)	60 ± 1	>99.9
**4**	Linker [Co(dcpgH)(dcpgH$_2$)Cl$_2$] (1 mM)	60 ± 3	>99.9
**5**	[{*meso*-tetra(4-carboxyphenyl)porphyrinato}cobalt(III)] chloride, CoTCPP (1 mM)	54 ± 2	>99.9
**6**	ZrCo-MOF (1 mg, 0.44 mM cobaloxime concentration)	35 ± 2	99.8

Summary of the reaction conditions used for the photocatalytic and control experiments. In a typical reaction, 2.0 mL of a C_2H_2 (≥99.5 vol.%)-purged solution containing catalyst, 2.5 mM $[Ru(bpy)_3]^{2+}$, 0.1 M BIH, 1.0 M TFE was irradiated at 450 nm (140 mW·cm^{-2}) for 4 h. Products were detected via GC-FID and experiments were performed in triplicates.

Supplementary References

[1] F. Arcudi, L. Đorđević, N. Schweitzer, S. I. Stupp, E. A. Weiss, *Nat. Chem.* **2022**, 14, 1007.
[2] G. N. Schrauzer, G. W. Parshall, E. R. Wonchoba, in *Inorg. Synth.*, Vol. 11 (Ed: W. L. Jolly), McGraw-Hill Book Company, Inc., 1968, 12.
[3] S. Roy, Z. Huang, A. Bhunia, A. Castner, A. K. Gupta, X. Zou, S. Ott, *J. Am. Chem. Soc.* **2019**, 141, 15942.
[4] L. S. Xie, S. S. Park, M. J. Chmielewski, H. Liu, R. A. Kharod, L. Yang, M. G. Campbell, M. Dinca, *Angew. Chem. Int. Ed.* **2020**, 59, 19623.
[5] a) T. Wakabayashi, K. Kamada, K. Sekizawa, S. Sato, T. Morikawa, J. Jung, S. Saito, *Organometallics* **2022**, 41, 1865; b) P. M. Stanley, J. Haimerl, C. Thomas, A. Urstoeger, M. Schuster, N. B. Shustova, A. Casini, B. Rieger, J. Warnan, R. A. Fischer, *Angew. Chem. Int. Ed.* **2021**, 60, 17854.
[6] J. Osswald, K. Kovnir, M. Armbruster, R. Giedigkeit, R. Jentoft, U. Wild, Y. Grin, R. Schlogl, *J. Catal.* **2008**, 258, 219.
[7] C. Cao, S. C. Fung, *Chem. Eng. Technol.* **2006**, 29, 307.
[8] M. J. Frisch, G. W. Trucks, H. B. Schlegel, G. E. Scuseria, M. A. Robb, J. R. Cheeseman, G. Scalmani, V. Barone, G. A. Petersson, H. Nakatsuji, X. Li, M. Caricato, A. V. Marenich, J. Bloino, B. G. Janesko, R. Gomperts, B. Mennucci, H. P. Hratchian, J. V. Ortiz, A. F. Izmaylov, J. L. Sonnenberg, Williams, F. Ding, F. Lipparini, F. Egidi, J. Goings, B. Peng, A. Petrone, T. Henderson, D. Ranasinghe, V. G. Zakrzewski, J. Gao, N. Rega, G. Zheng, W. Liang, M. Hada, M. Ehara, K. Toyota, R. Fukuda, J. Hasegawa, M. Ishida, T. Nakajima, Y. Honda, O. Kitao, H. Nakai, T. Vreven, K. Throssell, J. A. Montgomery Jr., J. E. Peralta, F. Ogliaro, M. J. Bearpark, J. J. Heyd, E. N. Brothers, K. N. Kudin, V. N. Staroverov, T. A. Keith, R. Kobayashi, J. Normand, K. Raghavachari, A. P. Rendell, J. C. Burant, S. S. Iyengar, J. Tomasi, M. Cossi, J. M. Millam, M. Klene, C. Adamo, R. Cammi, J. W. Ochterski, R. L. Martin, K. Morokuma, O. Farkas, J. B. Foresman, D. J. Fox, Wallingford, CT 2016.
[9] A. D. Becke, *J. Chem. Phys.* **1993**, 98, 5648.
[10] A. D. McLean, G. S. Chandler, *J. Chem. Phys.* **1980**, 72, 5639.
[11] C. Adamo, V. Barone, *J. Chem. Phys.* **1999**, 110, 6158.
[12] M. M. Francl, W. J. Pietro, W. J. Hehre, J. S. Binkley, M. S. Gordon, D. J. DeFrees, J. A. Pople, *J. Chem. Phys.* **1982**, 77, 3654.
[13] J. T. Muckerman, E. Fujita, *Chem. Commun.* **2011**, 47, 12456.
[14] M. Cossi, N. Rega, G. Scalmani, V. Barone, *J. Comput. Chem.* **2003**, 24, 669.
[15] A. Bondi, *J. Chem. Phys.* **2002**, 68, 441.
[16] S. Gnaim, A. Bauer, H. J. Zhang, L. Chen, C. Gannett, C. A. Malapit, D. E. Hill, D. Vogt, T. Tang, R. A. Daley, W. Hao, R. Zeng, M. Quertenmont, W. D. Beck, E. Kandahari, J. C. Vantourout, P. G. Echeverria, H. D. Abruna, D. G. Blackmond, S. D. Minteer, S. E. Reisman, M. S. Sigman, P. S. Baran, *Nature* **2022**, 605, 687.
